# Microstructure of Electrical Double Layers at Highly Charged States

**DOI:** 10.1021/jacsau.5c00508

**Published:** 2025-06-17

**Authors:** Zengming Zhang, Jun Huang

**Affiliations:** † Institute of Energy Technologies, IET-3: Theory and Computation of Energy Materials, 28334 Forschungszentrum Jülich GmbH, Jülich 52425, Germany; ‡ Theory of Electrocatalytic Interfaces, Faculty of Georesources and Materials Engineering, RWTH Aachen University, Aachen 52062, Germany

**Keywords:** electrical double layer, density-potential functional theory, differential double layer capacitance, short-range metal-solvent interaction, ion partial desolvation, electrochemical interface

## Abstract

While the traditional Gouy–Chapman–Stern (GCS) model can well describe the differential doublelayer capacitance (*C*_dl_) near the potential of zero charge with several empirical parameters, it is insufficient to capture the *C*_dl_ profile in a wide potential range and changes in the *C*_dl_ profiles with varying electrolyte cations, anions, and solvent, even for the atomistically smooth Mercury-solution interfaces. The extended data set of *C*_dl_ at mercury is then analyzed using modified semiclassical, density-potential functional theoretical (DPFT) models. Our analysis highlights the importance of potential-dependent short-range metal-solvent interactions and ion partial desolvation at highly charged surfaces. With the aid of the modified model, the impact of electrolyte cation, anion, and solvent on the EDL structure can be interpreted in an inherent framework. These insights gleaned from the mercury electrodes have crucial implications for the EDLs at gold, silver, and copper, which are usually highly charged in important electrocatalytic reactions like electrochemical CO_2_ reduction.

## Graphical abstract

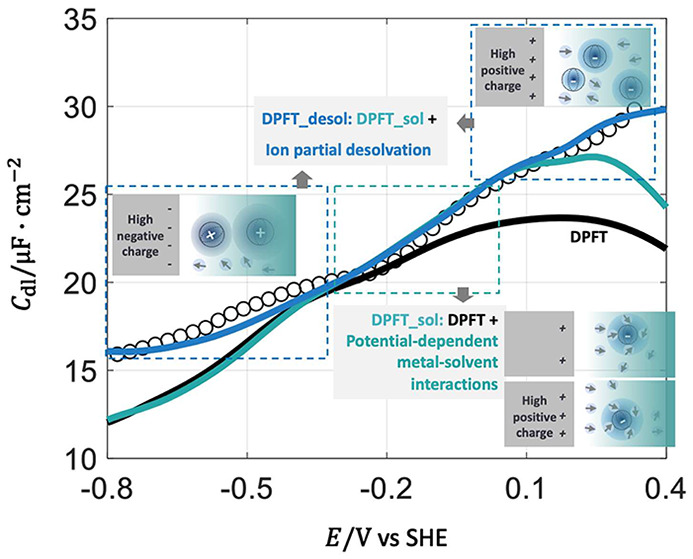

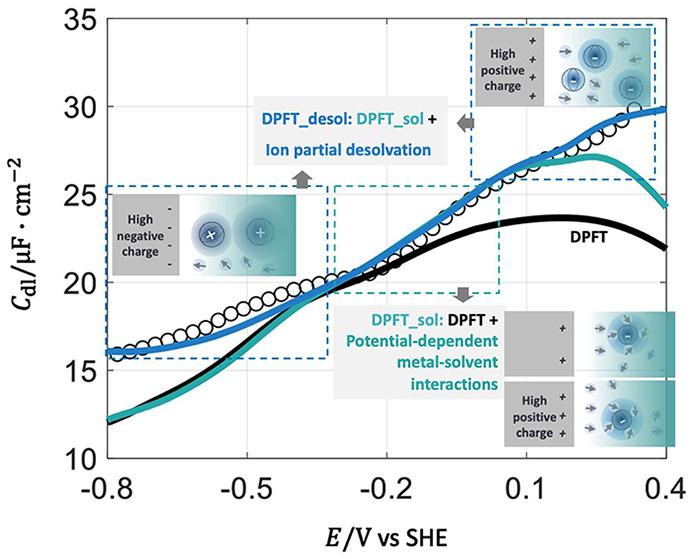

## Introduction

The interfacial region between a charged solid electrode and an electrolyte solution, known as the electrical double layer (EDL), is central to electrochemical energy conversion and storage.^1–4^ Symmetry breaking due to the presence of the electrode brings about characteristic distributions of species concentration, electrostatic potential, and dielectric permittivity in the adjacent electrolyte solution. These interfacial properties play a key role in tuning the activity and selectivity of electrocatalytic reactions.^1,4^ Recently, the effects of alkali metal cations on the activity and selectivity of electrocatalytic reactions are widely studied.^5–7^ For example, Goyal and Koper^8^ and Monteiro et al.^9^ demonstrated that compared with the case of Li^+^, K^+^ promotes the hydrogen evolution reaction (HER) at a gold electrode under alkaline conditions at low overpotentials, but inhibits the reaction at high overpotentials. However, the mechanisms behind the cation effects remain a topic of debate.^5,8,10,11^ The large consensus is that it is crucial to understand the structure and properties in EDL under realistic reaction conditions.

Contemporary understanding of the EDL is rooted in the classical Gouy–Chapman–Stern (GCS) model developed from the 1850s to the 1950s.^4^ In this model, the EDL comprises an inner layer between the metal surface edge and the central plane of rigidly aligned counterions (Helmholtz plane, HP), and an outer diffuse layer in which the ion distribution is determined by the competition between electrostatic force and thermal motion. The differential double layer capacitance, denoted as *C*_dl_, is a fundamental lumped parameter reflecting the EDL structure.^12^ The equivalent thickness of the EDL decreases due to counterion accumulation and then increases due to counterion overcrowding when the electrode potential, *E*_M_, deviates from the potential of zero charge (pzc, *E*_pzc_). A camel-shaped *C*_dl_ profile is very commonly observed in dilute solutions. The minimum point of the *C*_dl_ versus potential profile corresponds to the *E*_pzc_.^13,14^ The cathodic peak of the *C*_dl_ profile is usually attributed to cation overcrowding, while the anodic peak anion overcrowding. The larger the ion size, the lower the peak height. In highly concentrated solutions, the *C*_dl_ profile becomes bell-shaped, indicating a continuous increase in the EDL thickness as *E*_M_ moves away from *E*_pzc_, as elucidated by Kornyshev^15^ and also shown in Figure S1 in Supporting Information. We note more recent works reveal that the two peaks are ascribed mainly to orientational polarization of interfacial water molecules.^16,17^

Recent attention has largely focused on the EDL in the potential range near the pzc.^18^ For instance, the studies by Ojha et al. found that the Gouy–Chapman minimum is not observed in Pt(111)-HClO_4_ aqueous interfaces until the electrolyte concentration is decreased to 0.1 mM.^19^ The EDL at potentials beyond the vicinity of the pzc is much less studied, partly due to the difficulty in accurately measuring *C*_dl_ in a wide potential range. However, several important electrocatalytic reactions occur at potentials very negative of the pzc. For example, the onset potentials of HER on Pd(111), Pt(111), Ag(111) and Au(111) in 0.1 M aqueous solution with pH = 13 are about −0.87, −0.74, −1.37, and −1.32 V vs SHE scales, respectively,^20^ while the corresponding pzcs are 0.1, 0.3, −0.5, and 0.5 V_SHE_.^21^ The onset potential of CO_2_ reduction reaction on Cu(111) in 0.05 M H_2_SO_4_ solution (pH = 1) is around −0.86 V_SHE_,^22^ while the pzc of Cu(111) is around 0.7 V_SHE_.^23^ The onset potential of CO_2_ reduction reaction (CO_2_RR) on Au(111) in 0.1 M H_2_SO_4_ solution (pH = 3) is about −0.5 V_SHE_,^24^ around 1 V negative of its pzc.

The above analysis indicates that an improved understanding of the EDL at large (both cathodic and anodic) potentials referenced to the pzc is needed. For this purpose, we choose mercury as the model electrode because the *C*_dl_ profiles in an extended potential range exist for this electrode.^25,26^ In addition, the *C*_dl_ profiles have been measured in a wide parametric space of the electrolyte solution, including different cations like Na^+^ and K^+^, different weakly adsorbing anions like F^–^ and PF_6_^–^, and different solvent molecules like water and dimethyl sulfoxide (DMSO).

In the reminder of this paper, we start our analysis by addressing an obvious question: how good is the GCS model, with necessary modifications, for the mercury EDL in a wide potential range in various electrolyte solutions. Then, to further improve over the GCS model, we employ the recent density-potential functional theoretical (DPFT) approach,^27,28^ which has been employed to understand the *C*_dl_ profiles of Ag single crystals in aqueous solutions^27^ and Au single crystals in nonaqueous solutions.^29^ The DPFT approach integrates an orbital-free quantum mechanical description of the metal electrons and a classical statistical field description of the electrolyte solution, providing a computationally efficient description of the EDL.^27–29^ In achieving quantitative agreement between the DPFT model and experimental *C*_dl_ profiles, the DPFT model is improved by introducing two key physical phenomena that are missing in classical EDL models, namely, the dependency of short-range metal-solvent interactions on the electrode potential and the ion partial desolvation at highly charged surfaces. Leveraging a refined DPFT model that incorporates these two critical physical effects, we proceed to evaluate experimental *C*_dl_ profiles across a spectrum of ion concentrations within various electrolyte solutions. This analysis encompasses diverse types of cations, anions, and solvent molecules. By systematically considering these effects, the improved DPFT model constitutes an effective tool to understand the EDL structure under more realistic reaction conditions.

## Gouy–Chapman–Stern (GCS) Model

Based on the GCS model, the differential double layer capacitance can be considered as a series connection between the Helmholtz layer capacitance and the diffuse layer capacitance

1 $$\frac{1}{C_{\mathrm{dl}}}=\frac{1}{C_{\mathrm{GC}}}+\frac{1}{C_{H}}$$

where the diffuse layer capacitance, *C*_GC_, can be obtained from solving *the modified PB equation* considering the ion size effect.^15,30^ For detailed derivations, see the Supporting Information

$$\begin{array}{l} \frac{\partial^{2}U}{\partial X^{2}}=\frac{\sinh(U)}{1+\frac{\nu }{2}(\gamma_{c}e^{-U}+\gamma_{a}e^{U}-\gamma_{c}-\gamma_{a})}\\ C_{\mathrm{GC}}=\frac{\partial \sigma_{\mathrm{free}}}{\partial U_{\mathrm{HP}}}=-\frac{\partial }{\partial U_{\mathrm{HP}}}{\left(\frac{\partial U}{\partial X}\right)}_{X={\mathrm{HP}}^{+}} \end{array}$$ 2

where *U* is the electric potential with reference to the thermal potential $\frac{k_{B}T}{e_{0}}$ with *e*_0_ being the elementary charge, *k*_B_ the Boltzmann constant, and *T* the absolute temperature, and *U*_HP_ is the electric potential at the HP. *X* is the dimensionless coordinate with respect to the Debye length $\lambda_{D}=\sqrt{\frac{k_{B}T\epsilon_{s}^{b}\epsilon_{0}}{2{e_{0}}^{2}n_{i}^{b}}}$ with *n*_i_^b^ being the ion number density in bulk solution, and ϵ_s_^b^ the relative permittivity of solvent in bulk solution. ν = 2*a*_t_^3^ *n*_0_^b^ is the bulk volume fraction of solvated ions with *a*_t_ being the lattice size in lattice-gas model.^30–32^ $\gamma_{i}={\left(\frac{2r_{i}}{R_{s}}\right)}^{3}$ is the relative size of ions referenced to solvent with *r*_*i*_ being the radius of solvated ion and *R*_s_ the diameter of solvent. σ_free_ is the excess free surface charge density. *C*_H_ is the Helmholtz layer capacitance. As a historical note, Grahame^25,33^ determined the *C*_H_ versus electrode potential curve from the measured *C*_dl_ curve at ∼ 1 M NaF after correcting for the diffuse layer capacitance described by the Gouy–Chapman theory. He then used the obtained *C*_H_ in eq 1 to analyze the *C*_dl_ curves for other concentrations. Therefore, Grahame himself did not model the *C*_H_ curve. For this reason, Kornyshev, Spohr, and Vorotyntsev described the Grahame approach as semiempirical,^34^ named GCS_se. Different from this semiempirical approach, a primitive approach involves calculating *C*_H_ from ϵ_H_ and ϵ_0_ being the dielectric permittivity of the space between the HP and the metal surface and vacuum, respectively, and δ_H_ the distance between the HP and the metal surface. This model is referred to as GCS_pm. We implement both approaches and a comparison in terms of *C*_dl_ between the GCS models and experiments in Hg-100 mM NaF aqueous solution,^33^ as shown in Figure 1. The fitting process of the GCS models are shown in Figures S1 and S2 in Supporting Information. Additionally, we performed a sensitivity analysis by incorporating a field-independent dielectric decrement correction into the GCS model. The resulting analysis, presented in Figure S1­(e) in Supporting Information, shows that while the capacitance curve is slightly adjusted, the impact on the fitted parameters remains minimal across the studied concentration range. The GCS model results are calculated using the following model parameters: ϵ_H_ = 6, δ_H_ = 0.91 Å, *r*_–_ = 3 Å, *r*_+_ = 7.5 Å. The fitted value of permittivity ϵ_H_ is within the usual value between the 3 and 6 for the description of HP.^35^ The fitted δ_H_ is an effective value reflecting the closest distance that ions can approach the electrode surface. The solvated ion radius *r*_±_ takes into account the ion-solvation interactions. Notably, the fitted radius of solvated Na^+^ is larger than the value of 3 Å estimated by *ab initio* molecular dynamics (AIMD) simulations^36^ for Na^+^ with one layer of water. This suggests that the GCS model effectively considers more-than-one water layers in the solvation shell of Na^+^.

**Figure 1:**
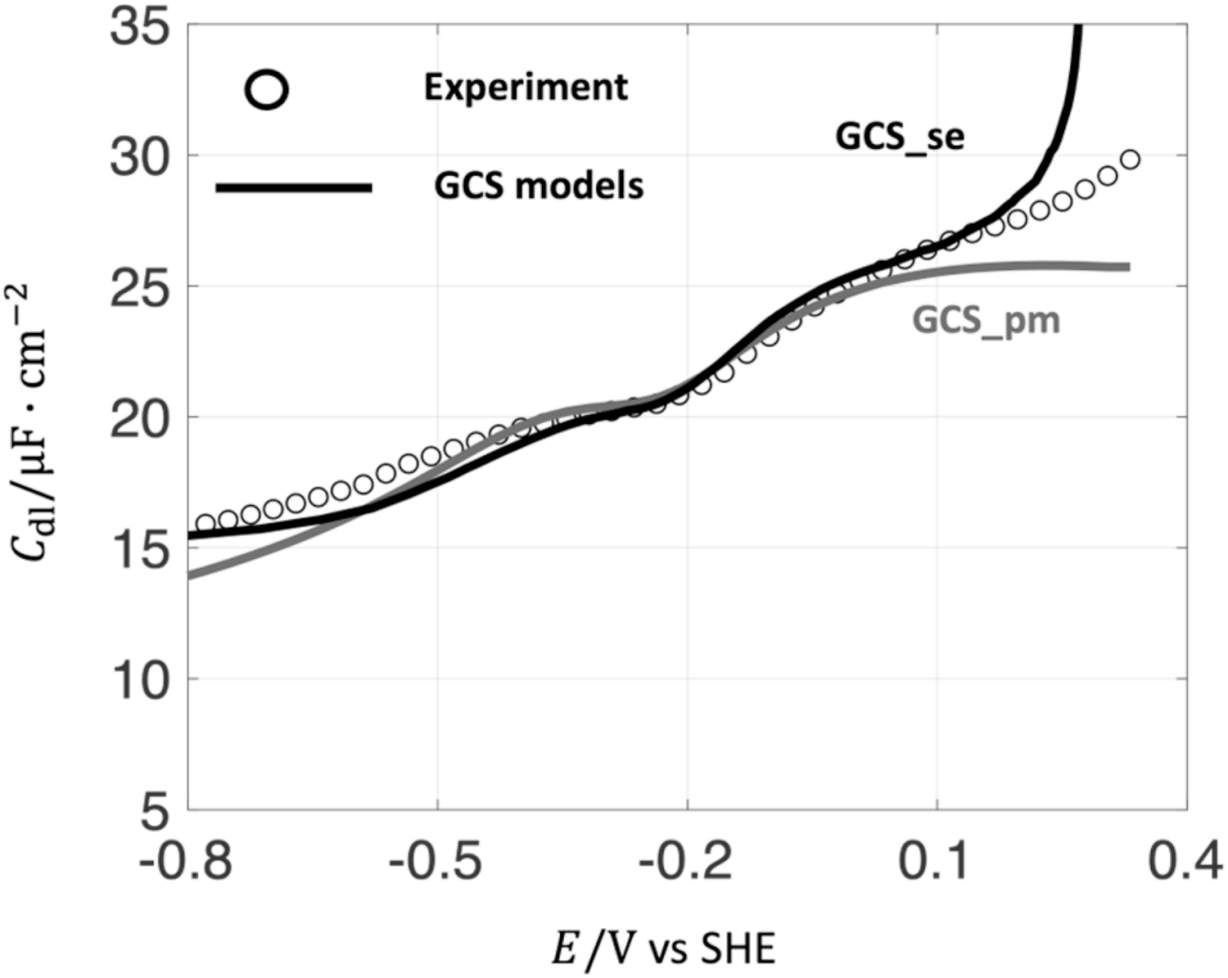
Comparison of *C*_dl_ between the experiment (circle) and the GCS models (solid line) in Hg-100 mM NaF aqueous solution. The GCS_se, considering the ion size effect, is compared with the primitive GCS model (GCS_pm). Fitted parameters are *ε*_H_ = 6, δ_H_ = 0.91 Å, *r*_–_ = 3 Å, *r*_+_ = 7.5 Å. Experimental data were reported by Grahame et al.^33^ The electrode potential is at the SHE scale.

In general, GCS_se and GCS_pm models can well reproduce the *C*_dl_ curves of the Hg-NaF aqueous interface near *E*_pzc_ = −0.22V_SHE_. The discrepancy between the GCS_pm model and the experimental data is observed at more positive potentials *E* > 0.1 V_SHE_, and more negative potentials *E* < −0.3 V_SHE_. The GCS_se model improves over GCS_pm in matching experimental data, but still overpredicts capacitance values at potentials beyond 0.2 V from the pzc. The divergence at more positive potentials is explained in the literature usually as the consequence of specifically adsorbed anions.^35,37–43^ For example, in the study of Wang et al.,^43^ a modified GCS model considering anion specific adsorption can neatly capture the whole *C*_dl_ profile in Ag(111)-NaF aqueous solution.^43^ Since the specific adsorption of F^–^ in the given potential range is rather weak and thus unlikely responsible for the observed divergence,^25^ we argue that there could be other causes.

Afterward, we systematically compare the *C*_dl_ curves between experiments and the GCS_pm model for different cations, anions, and solvent molecules,^33,40,44,45^ as shown in Figure 2. We exhibit the comparison at a high concentration, 100 mM, because the *C*_dl_ curves in more dilute solutions can be readily grasped once the more concentrated case is well understood. The solid points in Figure 2 represent the pzc, corresponding to the Gouy–Chapman minimum in dilute solutions. In Figure 2(a), the experimental *C*_dl_ profiles in the cathodic region are nearly identical for Na^+^ and K^+^, which have different solvation ion sizes. However, the GCS_pm model results in Figure 2(b) show significant differences. In the GCS_pm model, the cathodic peak of *C*_dl_ is smaller for Na^+^ than K^+^, as Na^+^ has a larger hydrated radius.^46^ In the anodic region, the two pieces of results calculated by the GCS_pm model are identical since the same anion, F^–^, is considered. We note that experimental results are taken from two separate studies,^33,44^ so we cannot exclude the possibility that the differences between the two experiments are just experimental errors, though the magnitude of differences exceeds the experimental error estimated by Grahame.^47^

**Figure 2:**
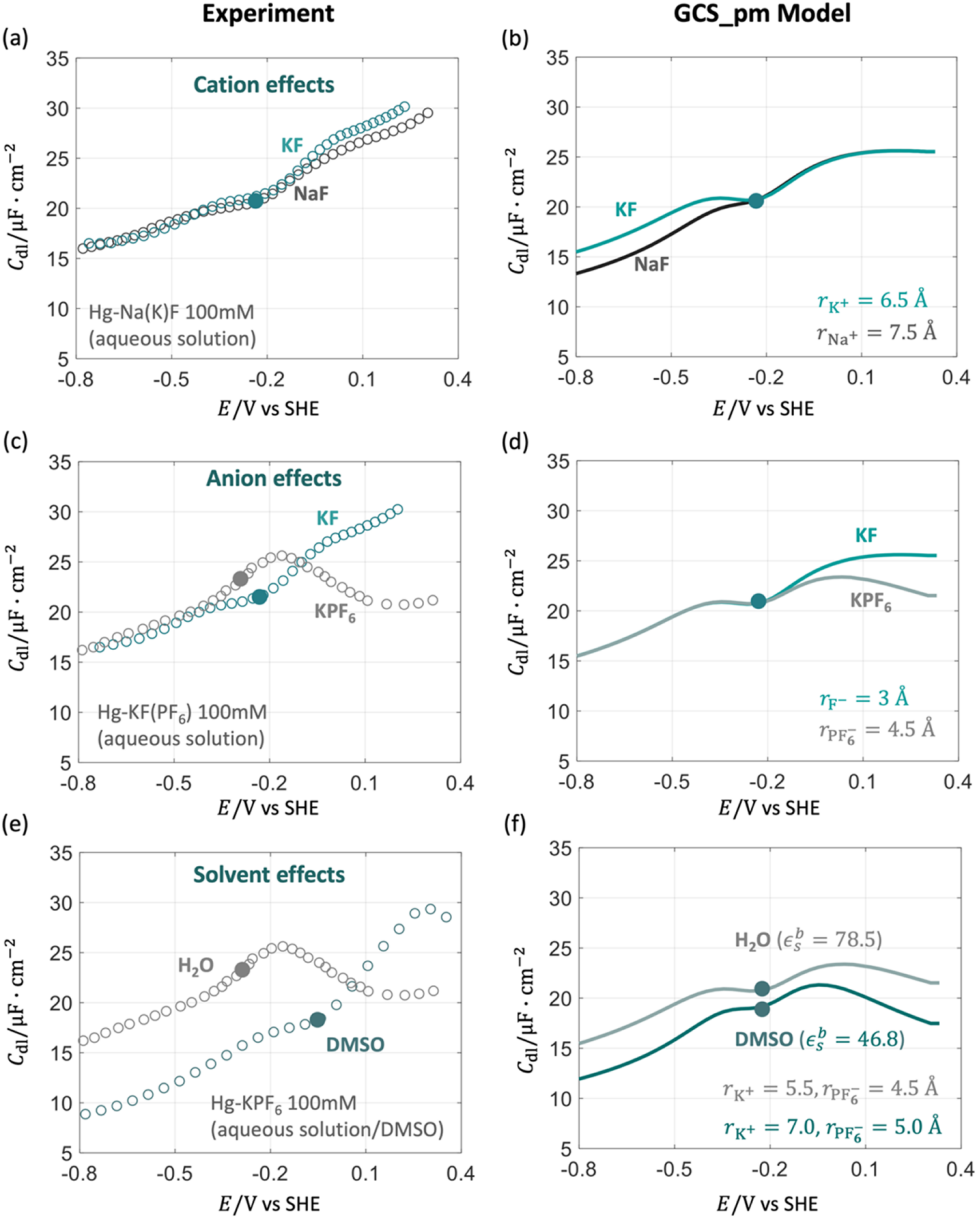
Comparison of *C*_dl_ between experimental results^33,40,44,45^ (a, c, e) and the GCS_pm model (b, d, f) for the EDL at the Hg-electrolyte solution interface with a concentration of 100 mM. (a, b) Effects of cations, Na^+^ and K^+^, on *C*_dl_, (c, d) effects of anions, PF_6_^–^ and F^–^, on *C*_dl_, and (e, f) effects of solvent molecules, water and DMSO, on *C*_dl_. Other parameters used in the GCS model are provided in Tables S1 and S2 in the Supporting Information. The solid points denote the potentials of zero charge.

The anion effects are examined in Figure 2(c). The experimental *C*_dl_ profiles in the cathodic region are nearly the same, as expected, since the same cation, K^+^, is used in both measurements. Consistent with the GCS_pm model, experimental data exhibits a smaller anodic *C*_dl_ peak for PF_6_^–^ than for F^–^, as PF_6_^–^ has a larger hydrated radius. However, the GCS models cannot account for the difference in the pzc. PF_6_^–^ has a more negative pzc compared to F^–.^ ^40^ Moreover, the rising trend at more anodic potentials for F^–^ is not captured by the GCS_pm model.

In Figure 2(e), we examine the effects of solvent molecules on the experimental *C*_dl_ profiles. Experiments show that the pzc is more positive in DMSO than H_2_O, which cannot be explained by the GCS_pm model. Moreover, it is interesting to note that the *C*_dl_ profiles intersect at around 0.05. At potentials negative of 0.05 V_SHE_, the experimental *C*_dl_ is smaller for DMSO than H_2_O, while the opposite trend is observed at potentials positive of 0.05 V_SHE_. On the contrary, the GCS_pm model gives a *C*_dl_ profile, which is constantly smaller for DMSO than H_2_O. The reasons for the magnitude difference are, at least, 2-fold. On the one hand, DMSO has a lower permittivity (ϵ_s_^b^ = 46.8) compared to H_2_O (ϵ_s_^b^ = 78.5). On the other hand, both K^+^ and PF_6_^–^ ions have larger solvated radii in DMSO than H_2_O, as shown in Figure 2(f).

In a word, the GCS_se and GCS_pm model efficaciously describe the *C*_dl_ in the potential region near the pzc. However, it is deficient to describe the *C*_dl_ profile far away from the pzc. The deficiency is more apparent when the electrolyte effects on the pzc and the *C*_dl_ are considered. Therefore, there is a clear need for an improved model for mercury’s EDL. As mentioned in the introduction, the DPFT will be employed as the model framework to analyze all of the above *C*_dl_ curves. Our proposed DPFT framework can be seen as an improved description of the complex behavior of *C*_H_ within a more detailed and physically motivated structure. It also goes beyond the GCS framework by incorporating a microscopic treatment of free and bound solvent molecules, potential-dependent metal-solvent short-range interactions, and partial desolvation of ions. In the next section, we will introduce three improvements to the DPFT model compared to our previous works.^27,29,48^

## DPFT Models

The framework and new modifications of the DPFT are introduced in this section. We realize that our previous DPFT model, denoted DPFT_pre, is deficient in describing the interfacial permittivity as it may go unrealistically higher than the permittivity of bulk water, see Figure 3(h) in ref 27 Herein, we refine the description of interfacial permittivity, following Dreyer et al.,^49^ by distinguishing free solvent molecules from those trapped in the solvation shell of ions, and further accounting for the dielectric screening capabilities of the trapped solvent molecules. This updated DPFT model is denoted DPFT. As to be rationalized in the subsequent analysis of experimental data, the DPFT model is further modified by introducing the potential dependence of short-range metal-solvent interactions, denoted the DPFT_sol model, and the partial desolvation of ions at highly charged surfaces, denoted the DPFT_desol model. A summary of features of the three DPFT models is given in Figure 3.

**Figure 3:**
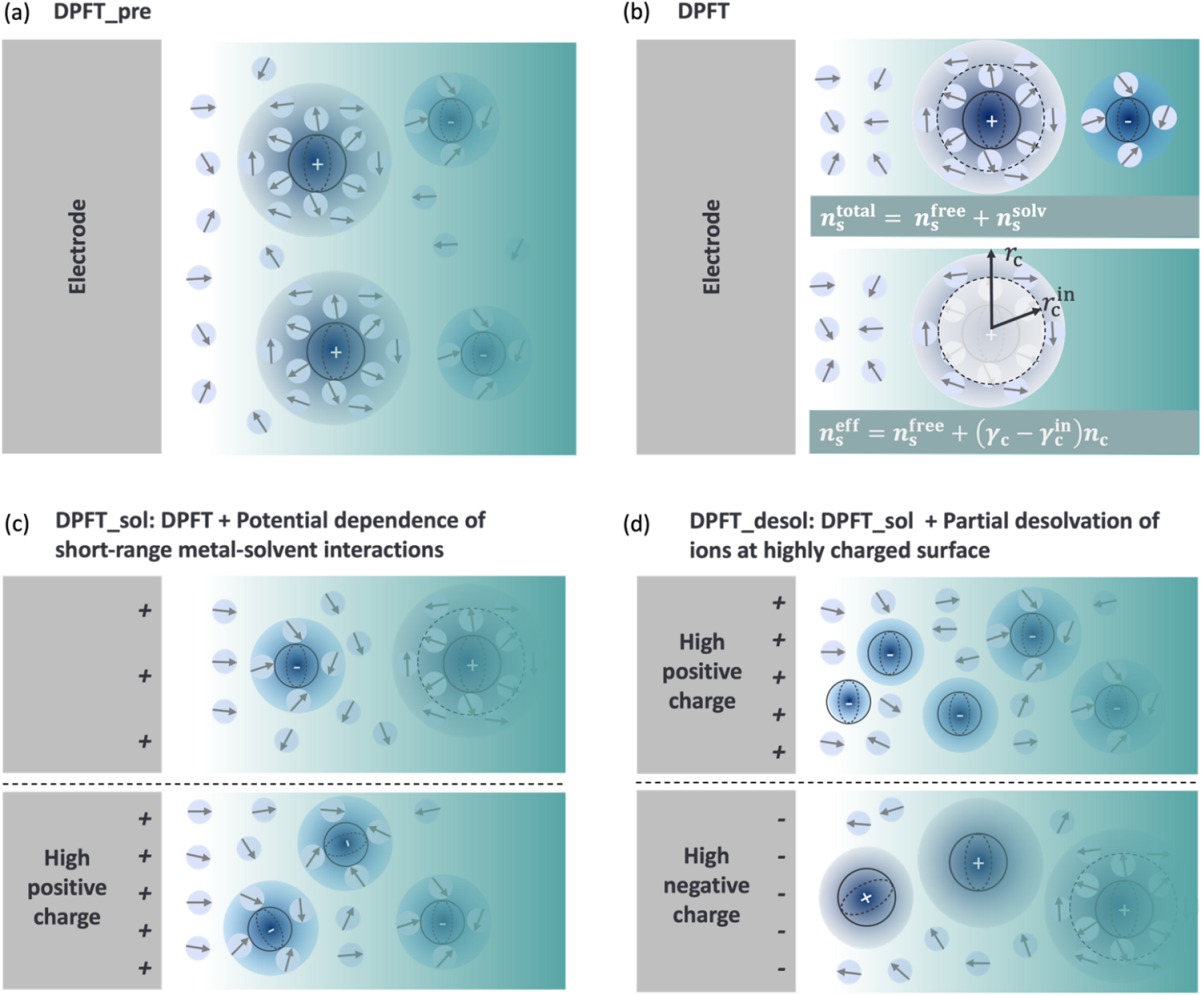
Schematic diagram of four versions of the DPFT. (a) DPFT_pre: the previous DPFT model.^27^ (b) DPFT: the solvent molecules are divided into free ones with a number density of *n*_s_^free^ and trapped ones with a number density of *n*_s_^solv^. (c) DPFT_sol: DPFT + the potential dependence of short-range metal-solvent interactions. (d) DPFT_desol: DPFT_sol + the partial desolvation of ions at highly charged surfaces.

In the DPFT, the volumetric density of the grand potential of the EDL is given by^27,28^

3 $$g=f_{\mathrm{qm}}\left[n_{e},\nabla n_{e}\right]+f_{c}\left[\phi ,\nabla \phi ,\left\{n_{i}\right\}\right]+f_{\mathrm{int}}\left[\phi ,\nabla \phi ,\left\{n_{i}\right\}\right]-\sum_{i}n_{i}{\mathrm{\mu ̃}}_{i}$$

where *f*_qm_ represents the internal energy of the electron gas, which is a functional of electron density *n*_*e*_ and its gradient ∇*n*_e_. *f*_c_ describes the classical interactions between charged particles, which is a functional of the densities of charged particles *n_i_* and electric potential ϕ and its gradient ∇ϕ. *f*_int_ describes the short-range interactions between electrolyte component *i* and the metal surface. μ*_~i_* is the electrochemical potential of component *i*. The governing equations of the EDL model can be obtained through variational analysis of the grand canonical potential.^27,28^ As detailed in the Supporting Information, the controlling equation for the electron density *n*_e_ in terms of the dimensionless electron density, *n̅*_e_ = *n*_e_ *a*_0_^3^, is expressed as

4 $$\mathrm{\nabla ̅}\mathrm{\nabla ̅}{\mathrm{n̅}}_{e}=\frac{20}{3}{\mathrm{n̅}}_{e}\frac{\omega }{\theta_{T}\omega -\theta_{\mathrm{XC}}}\left(\frac{\partial t_{\mathrm{TF}}}{\partial {\mathrm{n̅}}_{e}}+\frac{\partial u_{X}^{0}}{\partial {\mathrm{n̅}}_{e}}+\frac{\partial u_{C}^{0}}{\partial {\mathrm{n̅}}_{e}}-\frac{\left(e_{0}\phi +{\mathrm{\mu ̃}}_{e}\right)}{e_{\mathrm{au}}}\right)+\frac{\left(\theta_{T}\omega -\frac{4}{3}\theta_{\mathrm{XC}}\right)}{2{\mathrm{n̅}}_{e}\left(\theta_{T}\omega -\theta_{\mathrm{XC}}\right)}\cdot {\left(\mathrm{\nabla ̅}{\mathrm{n̅}}_{e}\right)}^{2}$$

with $\omega =\frac{2}{5}\pi^{5/3}3^{1/3}{\left({\mathrm{n̅}}_{e}\right)}^{1/3}$ . The overbar denotes variables and operators in the dimensionless form, for instance, *∇̅* = *a*_0_ ∇ with the Bohr radius, *a*_0_ = 0.529 Å as the reference length. $t_{\mathrm{TF}}=\frac{3}{10}{\left(3\pi^{2}\right)}^{2/3}{\left(n_{e}a_{0}^{3}\right)}^{5/3}$ is the Thomas-Fermi kinetic energy functional, $s=|\nabla n_{e}|/\left(2{\left(3\pi^{2}\right)}^{1/3}{\left(n_{e}\right)}^{4/3}\right)$ is the reduced density gradient, and θ_T_ and θ_XC_ are the gradient coefficients tuning the contribution of the semilocal term in kinetic energy and exchange-correlation energy, respectively. The term *e*_au_ *a*_0_^–3^ is used to transform the expression from atomic units to SI units, with the atomic energy $e_{\mathrm{au}}=\frac{e_{0}^{2}}{4\pi \epsilon_{0}a_{0}}=27.2\;\mathrm{eV}.$

On the electrolyte solution side, the radius of the solvated cations is inherited from the analysis based on the GCS_pm model in Figure 2. The large value suggests that solvated cations contain multiple solvent layers. In our previous work,^27,29,48^ all trapped solvent molecules were frozen and a small optical dielectric permittivity was used for them. In this work, we make a more reasonable assumption that the solvent molecules beyond the first layer of the solvent cations can also shield the electric field via orientational polarization. In this refined description, the solvent molecules are divided into free ones with a number density of *n*_s_^free^ and trapped ones with a number density of *n*_s_^solv^, as shown in Figure 3(b). Therefore, the total number density of solvent molecules *n*_s_^total^ is expressed as

5 $$n_{s}^{\mathrm{total}}=n_{s}^{\mathrm{free}}+n_{s}^{\mathrm{solv}},n_{s}^{\mathrm{solv}}=\left(\gamma_{c}+\gamma_{a}\right)n_{i}$$

where *n*_s_^total^ is the total number density of solvent molecules.

The number density of solvent molecules that can effectively shield the electric field is the sum of that of free solvent molecules and those trapped in the solvation shell beyond the first layer

6 $$n_{s}^{\mathrm{eff}}=n_{s}^{\mathrm{free}}+\left(\gamma_{c}-\gamma_{c}^{\mathrm{in}}\right)n_{c}$$

where $\gamma_{c}^{\mathrm{in}}={\left(\frac{2r_{c}^{\mathrm{in}}}{R_{s}}\right)}^{3}$ is the relative size of cations only with the first solvation layer, with a radius of *r*_c_^in^, which is estimated based on literature data.^36,50^

The controlling equation for the electric potential ϕ in the dimensionless form reads

7 $$-\mathrm{\nabla ̅}\left({\mathrm{\epsilon ̅}}_{\mathrm{op}}\mathrm{\nabla ̅}\mathrm{\phi ̅}+{\mathrm{n̅}}_{s}^{\mathrm{eff}}{\mathrm{p̅}}_{l}\kappa L\right)=\kappa \left(\left({\mathrm{n̅}}_{\mathrm{cc}}^{0}-{\mathrm{n̅}}_{e}\right)+\sum_{i=a,c}{\mathrm{q̅}}_{i}{\mathrm{n̅}}_{i}\left(r\right)\right)$$

where $\mathrm{\phi ̅}=\frac{e_{0}\phi }{k_{B}T}$ is the dimensionless electric potential, $\kappa =\frac{e_{0}^{2}}{k_{B}T\epsilon_{0}a_{0}}$ is a composite number of fundamental constants with ϵ_0_ being the dielectric permittivity of vacuum, and $L=\coth\left(\mathrm{p̅}\mathrm{E̅}\right)-{\left(\mathrm{p̅}\mathrm{E̅}\right)}^{-1}$ being the Langevin function. ${\mathrm{\epsilon ̅}}_{\mathrm{op}}=\frac{\epsilon_{\mathrm{op}}}{\epsilon_{0}}$ , $\mathrm{p̅}=\frac{p}{e_{0}a_{0}}$ and ${\mathrm{q̅}}_{i}=\frac{q_{i}}{e_{0}}$ are the dimensionless optical permittivity, dipole moment of solvent, charge of electrolyte ions, respectively. ${\mathrm{n̅}}_{\mathrm{cc}}^{0}={4N}_{\mathrm{Hg}}{\left(\frac{a_{0}}{a_{\mathrm{Hg}}}\right)}^{3}=0.57$ is the dimensionless charge density of metal cationic cores with *N*_Hg_ = 80 representing the total number of electrons of a mercury atom, and *a*_Hg_ = 4.365 Å is the lattice constant of Hg. We note that a unit cell contains four mercury atoms.^51^

The number density of electrolyte ions and free solvent molecules is described by a modified Boltzmann relationship^27^

8 $${\mathrm{n̅}}_{l}=\frac{\Theta_{l}}{1+\sum_{l=a,c,s^{\mathrm{free}}}^{N_{C}}n_{l}^{b}/n_{\max}\left(\Theta_{l}-1\right)}$$

where *n*_max_ = Λ_B_^–3^ = ∑_*l*=a,c,s_^free^ *n*_*l*_^b^γ_*l*_/(1 – χ_v_) is the maximum number density considering the inevitable presence of vacancies in the bulk solution, χ_v_. The thermodynamic factors Θ_*l*_ are given by

9 $$\Theta_{l}=\exp\left(-\beta \left(\delta \left(l\in M\right)q_{i}\phi -\delta \left(l\in S\right)\beta^{-1}\ln\frac{\sinh\left(\beta p_{l}\left|\nabla \phi \right|\right)}{\beta p_{l}\left|\nabla \phi \right|}+w_{l}\right)\right)$$

where β = (*k*_B_T)^−1^ is the inverse thermal energy. The Dirac function δ­(*l* ∈ *M*) is equal to one for cations and anions, and zero otherwise, δ­(*l* ∈ *S*) is equal to one for dipolar solvent molecules (*S*) and zero otherwise. *w*_*l*_, the short-range interactions between the metal surface and solution particles, are described using empirical potentials.^52^ Similar to a recent work,^53^ we use the repulsive part of the Morse potential to prevent ions and solvent from penetrating into the metal phase, written as,

10 $$w_{l}\left(\mathrm{r⃗}\right)=D_{\mathrm{ml}}\cdot \exp\left(-2\beta_{l}\left(d\left(\mathrm{r⃗}\right)-d_{\mathrm{ml}}\right)\right)$$

with *D*_ml_ being the well depth (*l* = a, c, s represent the anion, cation, and free solvent molecules, respectively), β_l_ a coefficient controlling the well width, *d*(*r⃗*) the distance from *r⃗* to the metal surface edge, and *d*_ml_ being the equilibrium distance between the particle and the metal surface. When *r⃗* is within the metal, *d*(*r⃗*) is negative and *w*_*l*_(*r⃗*) becomes very positive, meaning that solution particles have a negligible probability there. These parameters in eq 10 can be determined from the Kohn–Sham density functional theory (DFT) calculations. The binding energy of water on mercury according to DFT calculations is about 13.1 kcal/mol,^54^ namely, *D*_ms_ = 0.568 eV, and the distance between the water molecules and the metal surface is about 3.3 Å,^55^ namely, *d*_ms_ = 6.24*a*_0_.

The DPFT_sol model further considers the potential-dependent adsorption energy of solvent molecules. In the current framework, we allow the parameters of short-range metal-solvent interactions to vary with electrode potential. In a linear approximation, we assume

11 $$D_{\mathrm{ms}}=D_{\mathrm{ms}}^{0}+\alpha_{\mathrm{ms}}\left(E-E_{\mathrm{pzc}}\right)e_{0}$$

12 $$d_{\mathrm{ms}}=d_{\mathrm{ms}}^{0}-\beta_{\mathrm{ms}}\left(E-E_{\mathrm{pzc}}\right)e_{0}$$

where *D*_ms_^0^ and *d*_ms_^0^ are the well depth and the equilibrium distance between the solvent molecule and the metal surface at the pzc, respectively. The adsorption energy of solvent becomes larger with increasing electrode potential,^56–58^ which means that the solvent molecules can approach the metal surface to a closer distance, as shown in Figure 3(c). The dimensionless slopes α_ms_ and β_ms_ are to be determined from fitting the experimental *C*_dl_.

The DPFT_desol model improves over the DPFT_sol model by considering the partial desolvation of ion at highly charged surface, or in the GCS picture, the compression of the Stern layer.^59^ In the current model framework, this means that γ_i_ depends on the local electric field and becomes spatially varying. Specifically, γ_i_ will decrease near the metal surface with a strong electric field that can ‘liberate’ trapped solvent molecules from the solvation shell, as shown in Figure 3(d). Without losing generality, we assume a linear relationship

13 $$\frac{\gamma_{i}}{\gamma_{i}^{0}}=1-\zeta_{i}\left(\mathrm{\nabla ̅}\mathrm{\phi ̅}\right)$$

where γ_i_^0^ is the relative size of the solvated ion in solution bulk, ζ_i_ is a dimensionless coefficient indicating the degree of partial desolvation of ions. This parameter is to be estimated by fitting the DPFT_desol model with experimental *C*_dl_.

Currently, unknown model parameters are θ_T_, *ϵ̅*_op_ β_l_, *D*_ma(c)_, *d*_ma(c)_, which are determined by comparing model-based and experimental *C*_dl_ at Hg-NaF aqueous solution.^33^ Model parameters are listed in Tables S1, S3, and S4. Unsurprisingly, the model will improve the agreement with experimental data as it introduces 3 new tunable parameters, α_ms_, β_ms_, and ζ_i_. However, the large number of model parameters originates from the complex nature of many-body interactions in the EDL. Our purpose here is not only to fit the experimental data, but also, by achieving a decent agreement, to bring us a more detailed structure of the EDL.

The DPFT models can simulate the metal-solution interface under constant potential conditions.^27,28^ This capability is equivalent to adjusting the electrochemical potential of electrons, represented as *μ̃*_e_

14 $${\mathrm{\mu ̃}}_{e}=\mu_{e}-e_{0}\phi$$

with $\mu_{e}=\frac{\partial t_{\mathrm{TF}}}{\partial {\mathrm{n̅}}_{e}}+\frac{\partial u_{X}^{0}}{\partial {\mathrm{n̅}}_{e}}+\frac{\partial u_{C}^{0}}{\partial {\mathrm{n̅}}_{e}}$ being the chemical potential of a homogeneous electron gas. ϕ is the electric potential.

*μ̃*_*e*_ is related to the electrode potential *E*_M_ on the SHE scale according to,^60,61^

15 $$-{\mathrm{\mu ̃}}_{e}=e_{0}\left(E_{M}+4.44V\right)-e_{0}\chi_{s}^{v}$$

where χ_s_^v^ is the surface potential at the solution-vacuum interface.^62^

The governing equations, eq 4 and eq 7, are closed with the boundary conditions. The gradient of the electron density (∇̅*n̅*_e_) and the gradient of the electric potential (*∇̅ϕ̅*) are both set at zero in the bulk metal

16 $$\mathrm{\nabla ̅}{\mathrm{n̅}}_{e}=0,\mathrm{\nabla ̅}\mathrm{\phi ̅}=0$$

Similarly, in the bulk solution, the electron density (*n̅*_e_) and the electric potential (*ϕ̅*) are also set at zero

17 $${\mathrm{n̅}}_{e}=0,\mathrm{\phi ̅}=0$$

The key lumped property of the EDL, *C*_dl_, is calculated by differentiating the surface free charge σ_free_ with respect to electrode potential

Cdl=∂σfree∂EM=−e0∂σfree∂μ̃e=e02a02∂∂μ̃e∫dx̅(n̅c−n̅a)=e02a02∂∂μ̃e∫dx̅(n̅e−n̅cc0). 18

with $\sigma_{\mathrm{free}}=-\frac{e_{0}}{a_{0}^{2}}\int d\mathrm{x̅}\left({\mathrm{n̅}}_{c}-{\mathrm{n̅}}_{a}\right)=-\frac{e_{0}}{a_{0}^{2}}\int d\mathrm{x̅}\left({\mathrm{n̅}}_{e}-{\mathrm{n̅}}_{\mathrm{cc}}^{0}\right)$

## Results and Discussion

### Improved Agreement with Experiments from DPFT to DPFT_Desol

The improvement of the DPFT_desol model over the DPFT model is examined using the experimental *C*_dl_ in Hg-NaF aqueous solution.^33^ Figure 4 shows the comparison between experiments and the DPFT model. On the solution side, the ion radius *r*_*i*_, solvent diameter *R*_s_, and the bulk permittivity ϵ_s_^b^, are the same as those determined using the GCS model, as shown in Table S1. The fitting parameters are θ_T_ = 1.53, ϵ_®op_ = 3.70, β_l_ = 1, *D*_ma(c)_ = *D*_ms_/6, and *d*_ma(c)_ = 7.56*a*_0_ = 4 Å. Herein, the fitted θ_T_ has a large influence on the pzc and reflects the overall quantum mechanical interactions of all mercury electrons, so it is different from the commonly used value of 5/3 for single-electron systems.^63^ The fitted optical permittivity ϵ_®op_ is within the common range between 3 and 6.^35^ The value of β_l_, which corresponds to 0.53 Å^–1^, is close to the typical range of 0.4 and 1.1 Å^–1^ for this kind of system.^52^ *D*_ma(c)_ is smaller than *D*_ms_ because ions are nonspecifically adsorbed here. *d*_ma(c)_ is larger than *d*_ms_, meaning that the solvent can approach the metal surface to a closer distance than the ions.^56^

**Figure 4:**
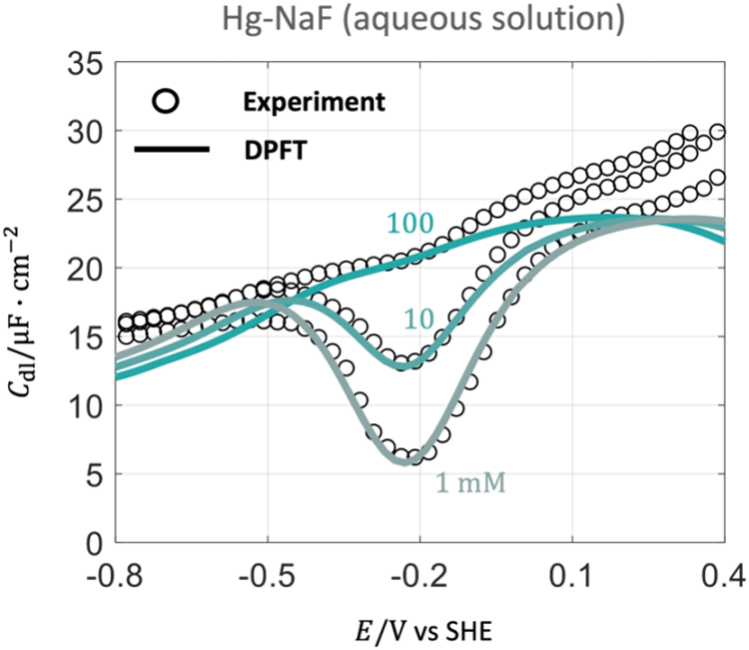
Comparison of *C*_dl_ between experimental results (circle) and DPFT model results (solid line) in Hg-NaF aqueous solution at varying ion concentrations. Calibrated parameters are θ_T_ = 1.53, ϵ_®op_ = 3.70, β_l_ = 1, *D*_ma(c)_ = *D*_ms_/6, and *d*_ma(c)_ = 7.56*a*_0_. Other parameters are given in Supporting Information. Experimental data are obtained from Grahame et al.^33^ The electrode potential is transformed to the SHE scale.

Focusing on *C*_dl_ in this section, we defer a discussion on detailed distributions of interfacial properties calculated by the DPFT model to the following section. The DPFT model can well reproduce the experimental *C*_dl_ profiles in the vicinity of the pzc at the three ion concentrations. However, beyond the vicinity of the pzc, the model results deviate noticeably from the experimental data. We show the deviation between the DPFT model and experimental data of 100 mM NaF in Figure S4 in the Supporting Information.

The improvement of DPFT_sol and DPFT_desol models over the DPFT model is shown in terms of the surface charging relationship, namely, σ_free_ versus *E* relationship, in Figure S6­(a,b) in the Supporting Information. The corresponding *C*_dl_ profiles are shown in Figures S6­(c) and 5(a). As shown in Figure S6­(a,c), the DPFT_sol model, introducing the potential-dependent short-range metal-solvent interactions, improves the agreement with the experimental data in the positively charged potential range of −0.2 < *E* < 0.1 V vs SHE. While the variation of *D*_ms_ and *d*_ms_ is small within the given electrode potential regions, as shown in Figure 5(c), but the improvement of *C*_dl_ in Figure S6­(c) is significant. The potential-dependent short-range metal-solvent interactions influence the free solvent density and the effective permittivity in the diffuse layer (4–10 Å), as depicted in Figure 8(a,b), leading to the observed improvement. These changes alter the electrostatic potential and ion density. A more detailed analysis will be provided in the following section.

**Figure 5:**
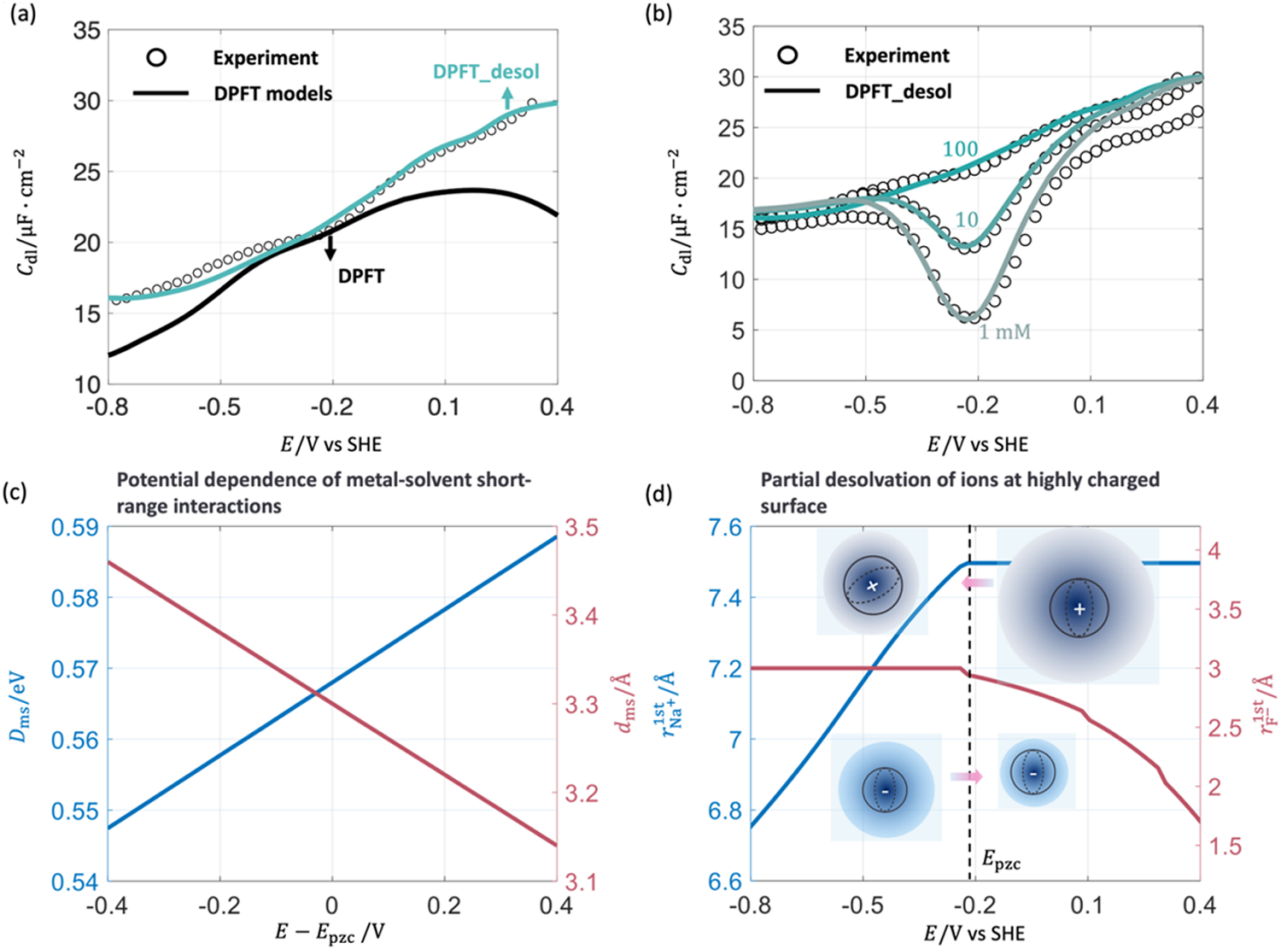
Improvement of the DPFT model by considering the potential-dependent short-range metal-solvent correlations in the DPFT_sol model and further considering the partial desolvation of ions in the DPFT_desol model. (a) Comparison of *C*_dl_ between the experiment and the DPFT models at 100 mM. (b) Comparison of *C*_dl_ between the experiment and the DPFT_desol model at varying ion concentrations. The electrode potential is referenced to the SHE. (c) *D*_ms_, *d*_ms_ change linearly with the electrode potentials with the fitting parameters α_ms_ = 0.05, β_ms_ = 0.40. The electrode potentials are referenced to the pzc. (d) Radii of solvated ion Na^+^ and F^–^, near the metal surface are as a function of the electrode potentials with fitting parameters ζ_Na^+^_ = 0.71, ζ_F^–^_ = 1.11.

However, the divergence between experimental data and the DPFT_sol model is still significant in highly positive and negative potential ranges. The DPFT_desol model, further introducing the partial desolvation of ions at highly charged surfaces, well reproduces the experimental σ_free_ and *C*_dl_ profiles in whole potential range, as shown in Figure S6­(b,c) in Supporting Information and Figure 5(a,b). A improved agreement is obtained between the DPFT_desol model and experimental results at varying ion concentrations, *c.f*. Figure 4 for the DPFT model. The fitted coefficients are α_ms_ = 0.05, β_ms_ = 0.40, ζ_Na^+^_ = 0.71, ζ_F^–^_ = 1.11. The key element of the improvement of the DPFT_desol model over the DPFT_sol model is the partial desolvation of electrolyte ions, leading to changes in the effective ion size with the electrode potential.

A two-dimensional diagram of the radius of the solvated ions, Na^+^ and F^–^, at different locations in the EDL under different electrode potentials is provided in Figure S8 in the Supporting Information. Figure 5(d) plots the radii of solvated ions closest to the metal surface, namely, *r*_Na^+^_^first^ and *r*_F^–^_^first^, as a function of electrode potential. *r*_Na^+^_^first^ decreases at more negative electrode potentials relative to the pzc because of partial desolvation, as described in eq 13, while *r*_Na^+^_^first^ is equal to its value in the bulk solution above the pzc. We note that the change in *r*_Na^+^_^first^ above the pzc has little influence on *C*_dl_ because cations are repelled from the positively charged surface. Similarly, *r*_F^–^_^first^ decreases at more positive electrode potentials relative to the pzc and equals its bulk value at potentials negative of the pzc. Partial desolvation effects increase the density of counterions in the EDL, as shown in Figure 8(e,f), leading to an increase in *C*_dl_. These insights are important to our understanding of the physical origins of the electrolyte effects on electrocatalytic reactions.^6,59,64^ For example, Li et al. employed *in situ* surface-enhanced infrared adsorption spectroscopy to probe the local electric field, revealing that cation dehydration amplifies the local electric field and facilitates the HER at Pt electrodes.^59^ A detailed analysis of electrolyte effects on the *C*_dl_ and their correlation with electrocatalytic reactions will be discussed in the final subsection. Moreover, we conducted an additional analysis by testing an alternative model, DPFT_desol_only, where the potential-dependent solvent-metal interactions were omitted while retaining the potential-dependent partial desolvation of ions effect, with parameters refitted accordingly. The comparison of the DPFT_desol_only model with experimental data across varying ion concentrations is shown in Figure S5. The results indicate that while the DPFT_desol_only model captures the general trends, it exhibits a notably worse agreement in the positive potential region compared to the full DPFT_desol model. Therefore, we conclude that including the potential dependence of short-range metal-solvent interactions is important for achieving quantitative agreement with experimental data, particularly in the positive potential region.

### Refined Microscopic Pictures of the EDL from DPFT to DPFT_Desol

The whole point of improving the fitting of *C*_dl_ lies in the rationale that a greater agreement with experimental *C*_dl_— a lumped parameter—would lead us to a more accurate spatially resolved, microscopic picture of the EDL. Like all inverse interference of higher-dimensional unknowns from lower-dimensional knowns, errors may slip into the process. The so-inferred higher-dimensional unknowns require independent validations; in the present case, the deduced EDL structure needs to be ultimately validated by *operando* measurements of the spatially resolved properties of the EDL, like the electrostatic potential distribution,^59,65,66^ which is beyond the scope of this work.

To set a baseline, we first look at the spatial distribution of interfacial properties calculated by the DPFT model at different electrode potentials. As the key improvement in the DPFT model compared to the DPFT_pre lies in the description of solvent molecules, we first examine the densities of various types of solvent and permittivity distribution in Figure 6. When the electrode potential deviates from the pzc, the density of free solvent decays from the bulk to the metal surface due to the steric repulsion of counterions. In contrast, the change in the effective solvent density is smaller due to the compensation of solvent molecules in the solvation shell of cations as expressed in eq 6. The effective permittivity, ϵ_s_^eff^, changes almost accordingly with the effective solvent density, as shown in Figure 6(c). A high ion concentration decreases *n*_s_^eff^ and, accordingly, the permittivity. The effect of ion concentration on the solvent density and permittivity at the pzc is shown in Figure S3 and compared with the experimental results.^67^ Additionally, *n*_s_^eff^ near the metal electrode is significantly higher than the bulk value, which agrees with AIMD simulations reported in the literature.^68^

**Figure 6:**
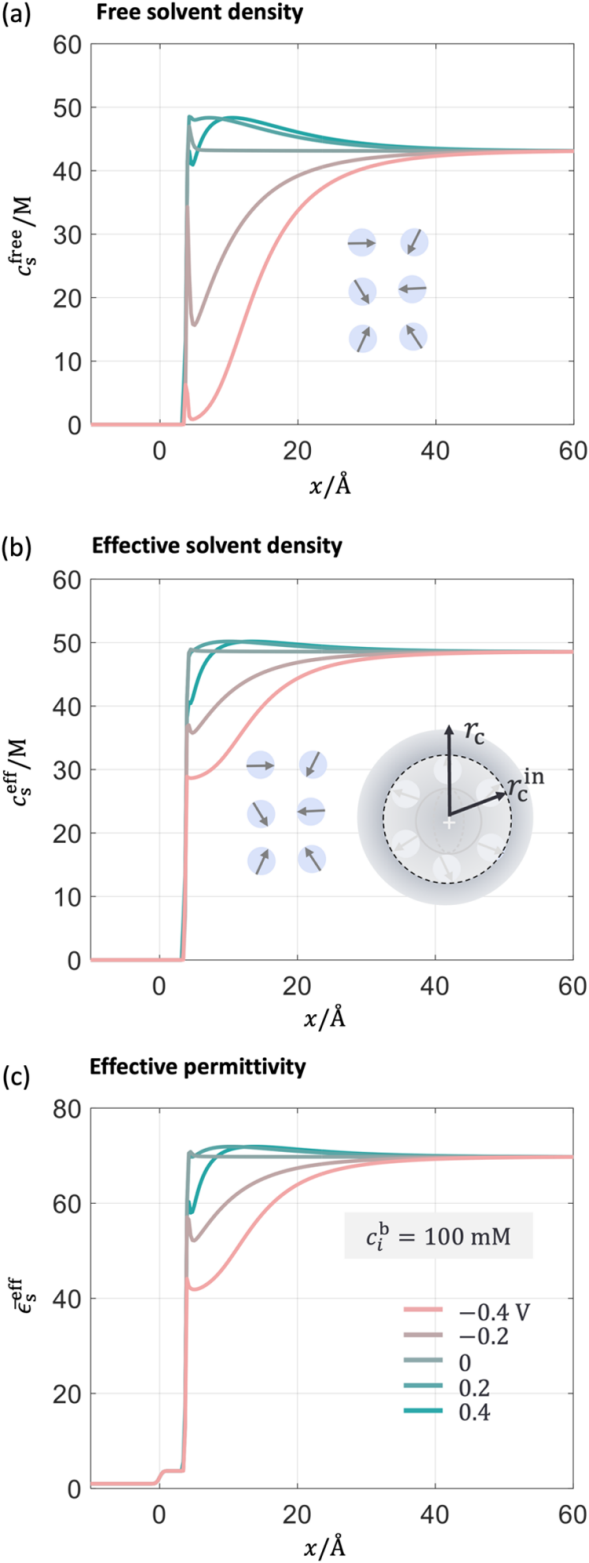
DPFT model results for Hg-NaF aqueous interface at 100 mM at five electrode potentials. Distribution of (a) free solvent density and (b) effective solvent density. (c) Distribution of the effective permittivity from the metal bulk to the solution bulk. The position *x* = 0 represents the metal edge and the electrode potential is referenced to the pzc.

Other interfacial properties, including distribution of cation and anion density *n*_a(c)_, electron density *n*_e_, and electrostatic potential ϕ are shown in Figure 7. As expected, when the metal electrode is negatively charged, cations are attracted to and anions repelled from the metal surface due to the long-range electrostatic interactions. The opposite occurs at a positively charged surface, as shown in Figure 7(a,b). These phenomena are already known from the classical GCS model.^30,69,70^ The electron tail stretches out more at more negative electrode potentials, as shown in the inset of Figure 7(c,d), which are consistent with the calculation from the jellium model.^71,72^ The electron density distribution, which is potential dependent and mediated by solvent properties, is important for understanding the electrolyte effects on pzc,^73,74^ as discussed in reference.^29^

**Figure 7:**
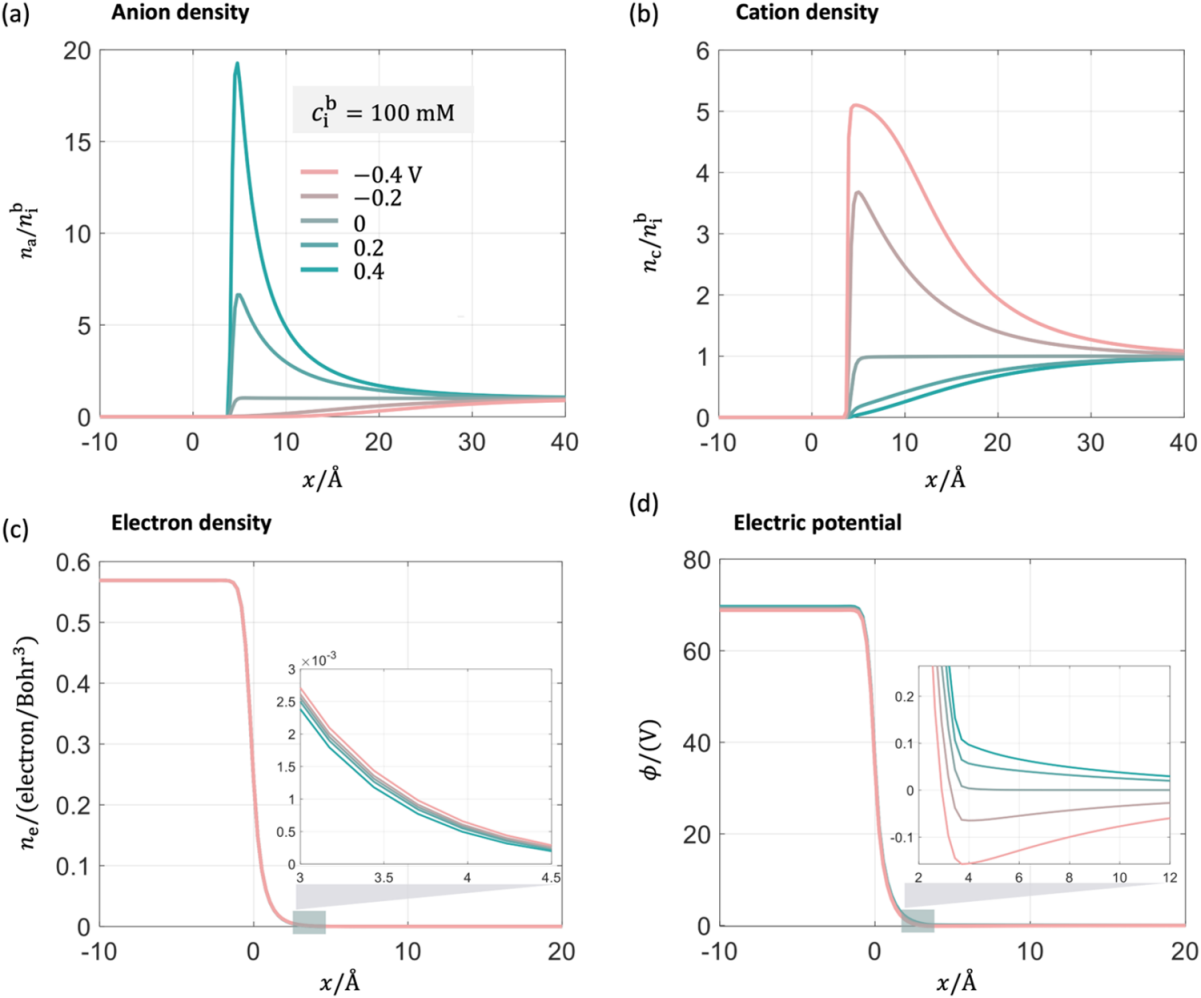
DPFT model results at 100 mM at five electrode potentials. The distribution of (a) anion and (b) cation density normalized to their values in the solution bulk. The distribution of (c) electron density *n*_e_, (d) electrostatic potential ϕ. The position *x* = 0 represents the metal edge and the electrode potential is referenced to the pzc.

Figure 8(a,b) illustrate that the potential-dependent short-range metal-solvent interactions introduced in the DPFT_sol model make the free solvent density and effective permittivity in the diffuse layer (4–10 Å) smaller at the positive potentials. These interactions have a minimal effect in the negative potential region. As a result, the electrostatic potential curve on the solution side rises, as shown in the inset of Figure 8(c), attracting more anions toward the metal surface, as depicted in Figure 8(d). This increased anion density leads to an increase in σ_free_ and *C*_dl_ in the positive potential region. Further improvements are seen in the DPFT_desol model, which incorporates the partial desolvation of ions at a highly charged surface. This model accounts for the increased density of interfacial cations and anions due to the reducing size effects, as shown in Figure 8(e,f), leading to a pronounced increase of σ_free_ and *C*_dl_.

**Figure 8:**
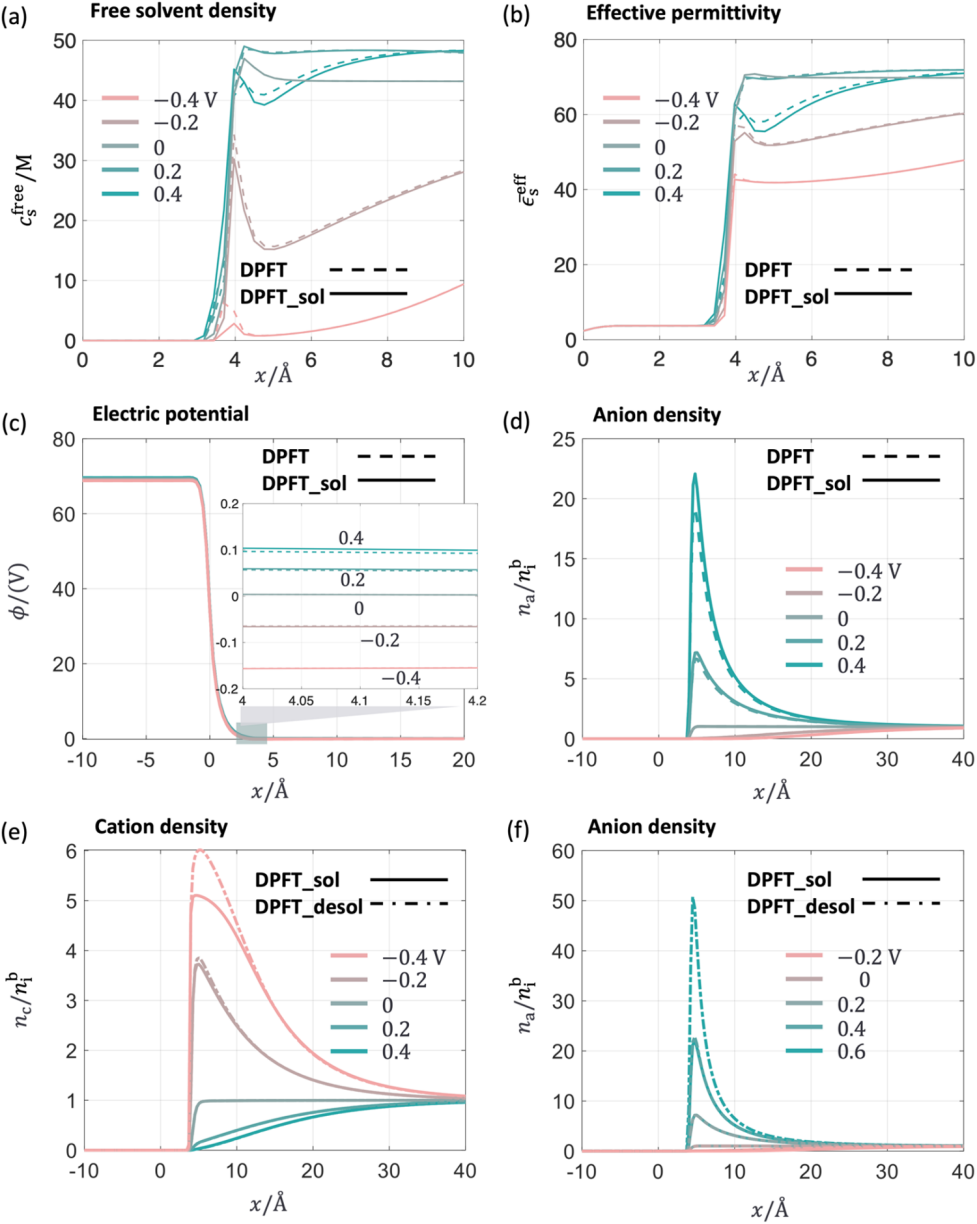
Interfacial structure of Mercury’s EDL at a concentration of 100 mM at five electrode potentials referenced to the pzc. Comparison of the distribution of (a) free solvent density, (b) the effective permittivity, (c) electric potential ϕ, and (d) anion density between the DPFT (dashed lines) and the DPFT_sol (solid lines) models. Comparison of the distribution of (e) cation density and (f) anion density between the DPFT_sol (solid lines) and the DPFT_desol (solid-dot lines) model. The position *x* = 0 represents the metal edge.

### Electrolyte Effects on the EDL

In this section, we extend the modified DPFT model to various electrolyte compositions. Specifically, we compare the DPFT, DPFT_sol, and DPFT_desol models with experimental *C*_dl_ curves for different electrolyte solutions containing various cations, anions, and solvent molecules. The model parameters are listed in Tables S3 and S4. When changing from one electrolyte to another, only a small set of parameters related to the electrolyte are varied, while other parameters remain unchanged. For instance, parameters describing the partial desolvation of ions are the same for the same ion in the same solvent.

As shown in Figure S6­(d–f) in Supporting Information, the DPFT_desol model significantly improves the agreement with experimental data of *C*_dl_ for KF aqueous solution, KPF_6_ aqueous solution, and KPF_6_ in DMSO, respectively. For each of the three electrolyte solutions, a comparison between the DPFT_desol model and experimental data at different ion concentration effects is shown in Figure 9(a–c), respectively. The decent agreement between the DPFT_desol model and experimental *C*_dl_ profiles across different electrolyte compositions lends credence to its effectiveness in describing the influence of electrolyte composition on the EDL of Mercury, which is detailed in the following.

**Figure 9:**
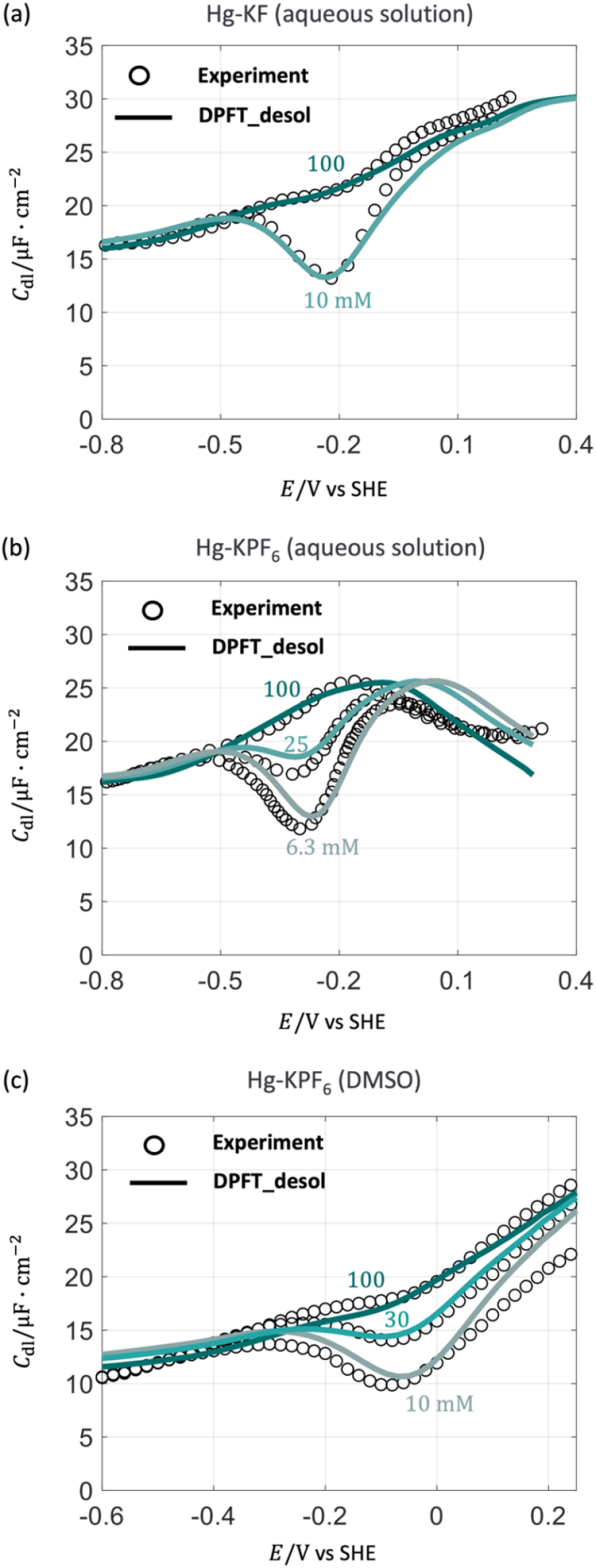
Extension of the improved DPFT model to different electrolyte compositions. Comparison of *C*_dl_ between the refined DPFT_desol model and experimental results for (a) Hg-KF aqueous solution reported by Schiffrin,^44^ (b) for Hg-KPF_6_ aqueous solution reported by Baugh and Parsons^40^ and (c) for Hg-KPF_6_ DMSO solution reported by Payne^45^ at different ion concentrations, respectively. Model parameters are provided in Table S4. The electrode potential is on the SHE scale.

The experimental *C*_dl_ curves for different cations, Na^+^ and K^+^, are compared with the DPFT_desol model at a concentration of 100 mM in Figure S7­(a) in Supporting Information. Though Na^+^ has a larger solvated size than K^+^,^46^ the *C*_dl_ curves in the negative potential region nearly overlap. This anomalous cation effect is captured by the DPFT_desol model, which accounts for cations undergoing different degrees of partial desolvation. Figure 10 shows the radii of solvated cations, Na^+^ and K^+^, near the metal surface as a function of the electrode potential, described by eq 13 with fitting parameters ζ_Na^+^_ = 0.71 and ζ_K^+^_ = 0.34. In comparison, solvated Na^+^ undergoes a greater degree of desolvation than K^+^ near the metal surface, which could be attributed to the stronger polarization effect of Na^+^ toward water molecules of the solvation shell of a neighboring Na^+^. This phenomenon can be validated using *in situ* surface-enhanced infrared adsorption spectroscopy.^59^

**Figure 10:**
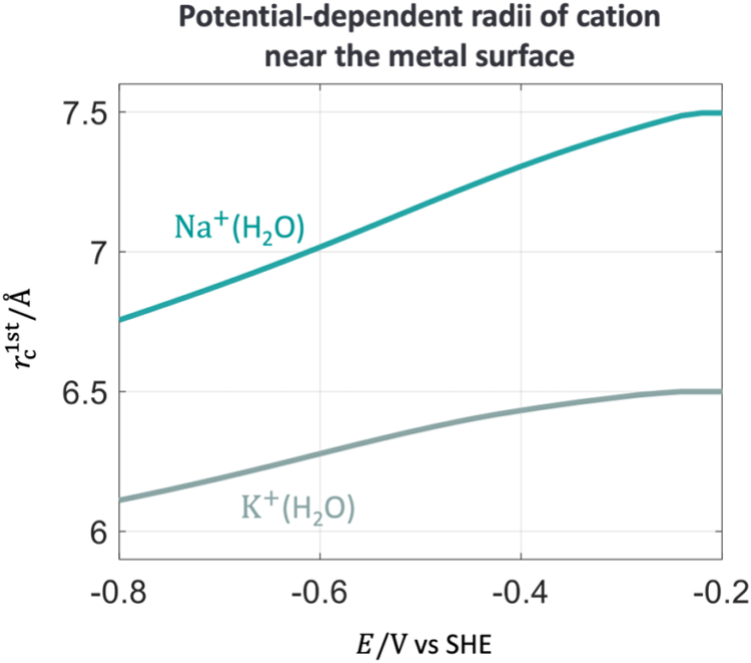
Radii of solvated ions, Na^+^(H_2_O) and K^+^(H_2_O), near the metal surface as a function of electrode potential. The calibrated parameters are ζ_Na^+^_ = 0.71 and ζ_K^+^_ = 0.34. Other parameters are provided in the Supporting Information. The solid point represents the pzc. The electrode potential is on the SHE scale.

Next, we analyze the experimental *C*_dl_ curves for different anions, F^–^ and PF_6_^–^, which are fitted with the DPFT_desol model in Figure S7­(b) in Supporting Information. The effect of anions on the pzc is already captured in the DPFT model, as shown in Figure 11(a), with fitting parameters *d*_mF^–^_ = 4 Å and *d*_mPF_6_^–^_ = 3.2 Å, respectively. These parameters suggest that the short-range metal-anion interactions may be stronger for PF_6_^–^ than F^–^, potentially causing the pzc to shift to a more negative value^27,29^ (*E*_pzc_ = −0.30 V_SHE_). Sundararaman et al.^52^ have calculated the metal-anion interactions for F^–^ on Ag(111), while a comparative calculation of PF_6_^–^ and F^–^ on mercury electrodes is missing.

**Figure 11:**
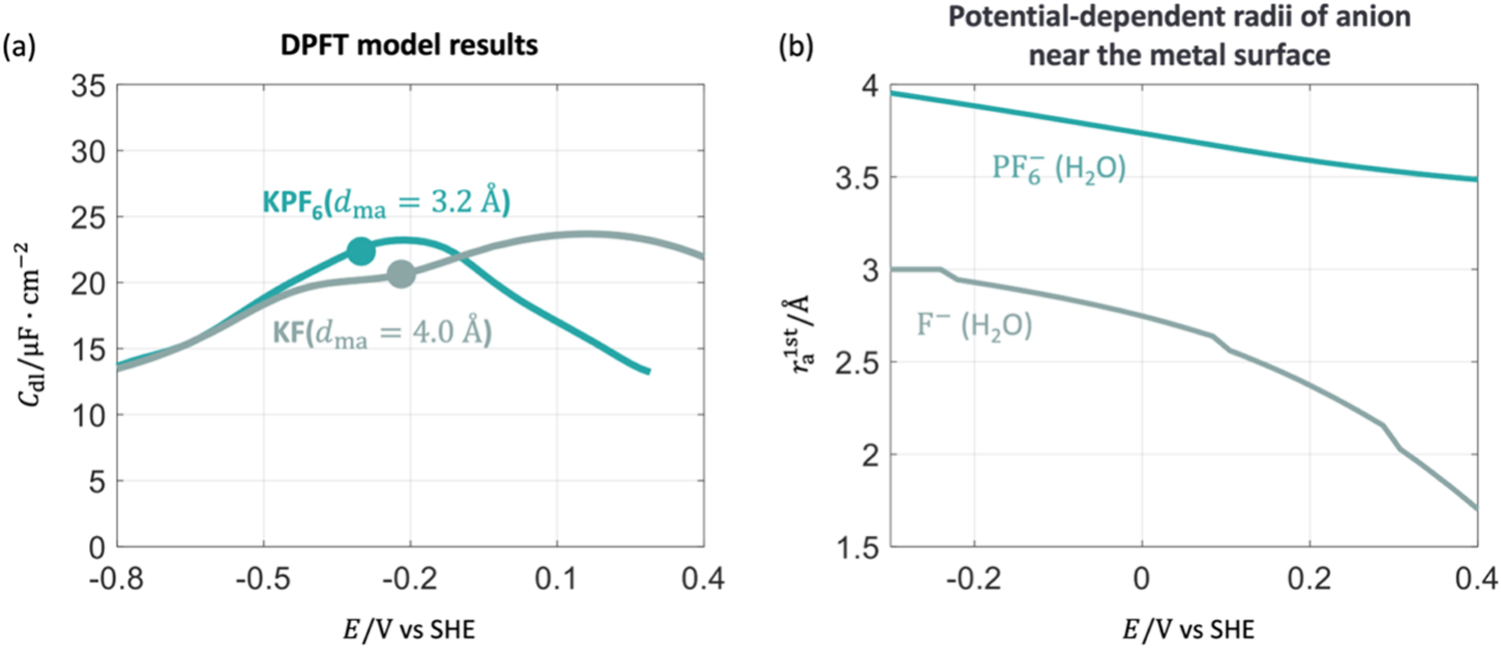
Effects of anions, PF_6_^–^ and F^–^, on *C*_dl_. (a) *C*_dl_ results obtained from the DPFT model. (b) Radii of solvated ions, F^–^(H_2_O) and PF_6_^–^(H_2_O), near the metal surface as a function of electrode potentials. The calibration parameters are *d*_mF^–^_ = 4 Å, *d*_mPF_6_^–^_ = 3.2 Å, ζ_F^–^_ = 1.11 and ζ_PF_6_^–^_ = 0.05. Other parameters are provided in the Supporting Information. The solid point represents the pzc. The electrode potential is on the SHE scale.

The influence of anions on the shape of *C*_dl_ can be understood through the variable radii of solvated anions. As depicted in Figure 11(b), the radii of solvated anions, F^–^ and PF_6_^–^, near the metal surface change with the electrode potential, described by eq 13 with fitting parameters ζ_F^–^_ = 1.11 and ζ_PF_6_^–^_ = 0.05, respectively. The DPFT_desol model analysis suggests that F^–^ undergoes a greater degree of desolvation than PF_6_^–^ at positive potentials. Nevertheless, *r*_F^–^_ remains smaller than *r*_PF_6_^–^_, resulting in an earlier capacitance peak for PF_6_^–^.

Finally, we study the solvent effects on the experimental *C*_dl_ curves, specifically, by comparing water and DMSO. In both solvents, KPF_6_ is used as the salt. The results calculated with the DPFT_desol model are presented in Figure S7­(c) in Supporting Information. As regards DMSO, its lower bulk and optical permittivity, along with a smaller metal-solvent equilibrium distance, shift the pzc positively (*E*_pzc_ = −0.05 V_SHE_), as shown in the DPFT model in Figure 12(a). The fitting parameters are *ϵ̅*_op_^H_2_O^ = 3.70, *d*_mH_2_O_^0^ = 3.3 Å and *ϵ̅*_op_^DMSO^ = 2.74, *d*_mDMSO_^0^ = 2.8 Å, respectively. The solvent effects on the pzc have been discussed in detail in a previous study.^29^

**Figure 12:**
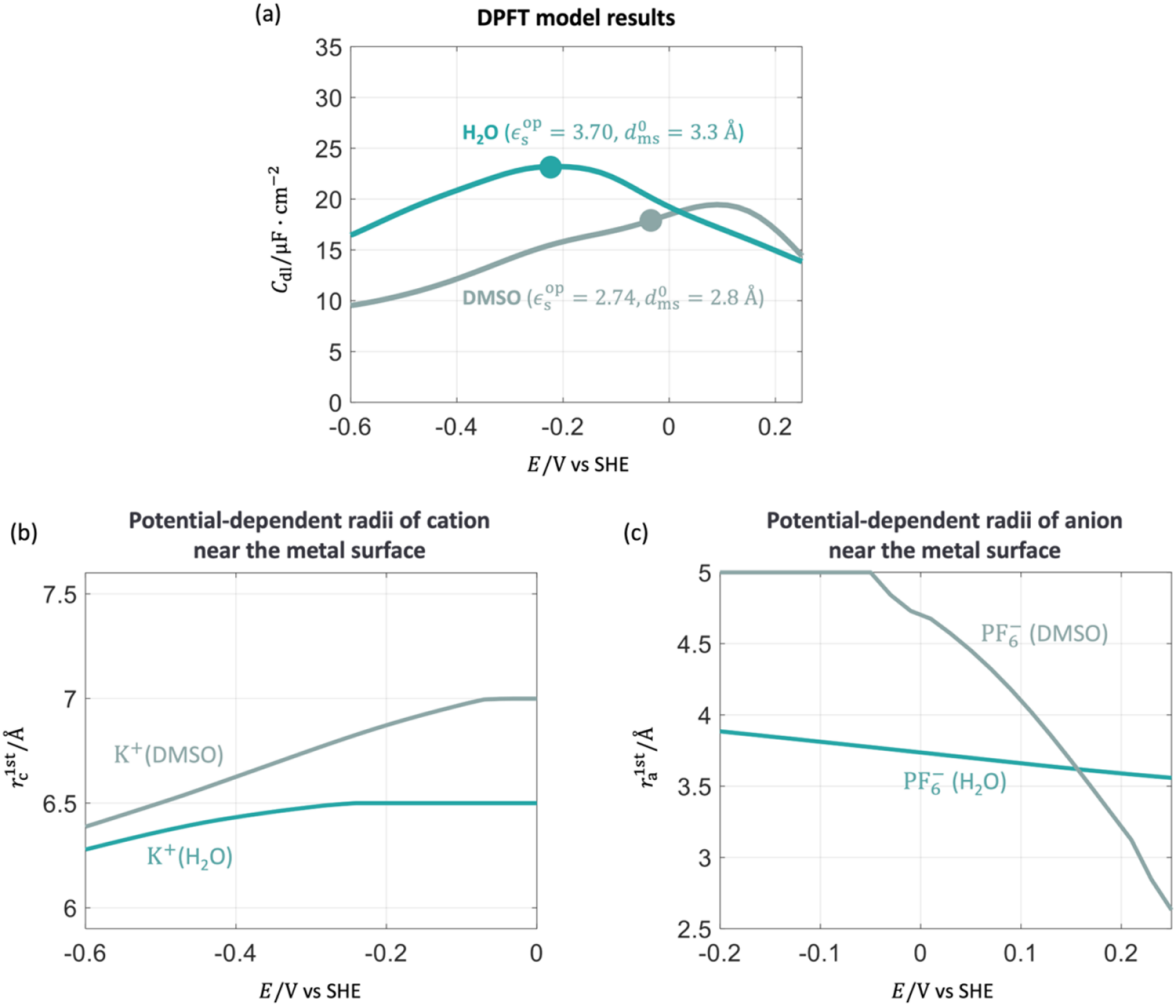
Effects of solvent molecules, water and DMSO, on *C*_dl_. (a) *C*_dl_ results obtained from the DPFT model. (b) Radii of solvated cations, K^+^(H_2_O)­and K^+^(DMSO), near the metal surface as a function of electrode potentials. (c) Radii of solvated anions, PF_6_^–^(H_2_O) and PF_6_^–^ (DMSO), near the metal surface as a function of electrode potentials. The calibration parameters are *ϵ̅*_op_^DMSO^ = 2.74, *d*_mDMSO_^0^ = 2.8 Å, α_mDMSO_ = 0.05, β_mDMSO_ = 0.15, ζ_K^+^_^H_2_O^ = 0.34, ζ_K^+^_^DMSO^ = 0.35, ζ_PF_6_^–^_^H_2_O^ = 0.05, and ζ_PF_6_^–^_^DMSO^ = 2.35. Other parameters are provided in the Supporting Information. The solid point represents the pzc. The electrode potential is on the SHE scale.

The effects of solvent molecules on the shape of *C*_dl_ are analyzed by examining the interfacial radii of solvated ions, as shown in Figure 12(b,c). Figure 12(b) presents the interfacial radii of solvated K^+^ in water and DMSO as a function of electrode potential. These radii are described by eq 13 with fitting parameters ζ_K^+^_^H_2_O^ = 0.34 and ζ_K^+^_^DMSO^ = 0.35, respectively, indicating that solvated K^+^ undergoes a greater degree of desolvation in DMSO at negative potentials compared to water. Furthermore, *r*_K^+^_^H_2_O^ remains smaller than *r*_K^+^_^DMSO^, resulting in a higher *C*_dl_ profile for water compared to DMSO at negative potentials. Similarly, Figure 12(c) shows the interfacial radii of solvated PF_6_^–^ in water and DMSO as a function of electrode potential. These radii are described by eq 13 with ζ_PF_6_^–^_^H_2_O^ = 0.05 and ζ_PF_6_^–^_^DMSO^ = 2.35, respectively, showing that solvated PF_6_^–^ undergoes greater desolvation in DMSO at positive potentials compared to water. Initially, *r*_PF_6_^–^_^H_2_O^ is smaller than *r*_PF_6_^–^_^DMSO^; however, as the potential increases, this trend reverses, causing *C*_dl_ profile in water to be higher initially but lower than that in DMSO at higher potentials. These desolvation behaviors may be attributed to the higher solvation energy of ions in aqueous solution compared to organic solvent, as discussed in prior studies.^73,74^

## Conclusions

In this study, we revisited the electrical double layer (EDL) at mercury electrodes, comparing the classical Gouy–Chapman–Stern (GCS) model and various semiclassical density-potential functional theoretical (DPFT) models in terms of describing the differential double layer capacitance (*C*_dl_). While the GCS model performs well near the potential of zero charge (pzc), it is deficient to capture the *C*_dl_ profiles in a more extended potential range, where several critical electrocatalytic reactions occur, and the electrolyte effects on the pzc and the Helmholtz capacitance (*C*_H_).

We aim at complementing the understanding based on the GCS model using a semiclassical model based on DPFT, which integrates an orbital-free quantum mechanical description of the electrode with a classical statistical field description of the electrolyte. We refine the description of interfacial permittivity by distinguishing free solvent molecules from those trapped in the solvation shell of ions, and by further incorporating the dielectric screening capabilities of the trapped solvent molecules. In addition, the potential-dependence of short-range metal-solvent interactions and the partial desolvation of ions at highly charged surfaces are incorporated into the DPFT model, termed DPFT_desol.

Comparisons between the DPFT_desol model and experimental *C*_dl_ profiles reveal the importance of potential-dependent short-range metal-water interactions in accurately predicting the upward-tilted *C*_dl_ profiles observed experimentally. Additionally, accounting for the partial desolvation of ions significantly improves the model’s alignment with experimental data, particularly at highly charged states.

The DPFT_desol model is further extended to various electrolyte compositions, successfully reproducing experimental *C*_dl_ profiles for different cations, anions, and solvent molecules. This highlights the robustness and generality of the DPFT approach. Detailed analysis of electrolyte effects on *C*_dl_ provides valuable insights that could be informative to understand the EDL at copper and other highly charged electrodes used in critical electrocatalytic reactions, such as CO_2_ reduction reactions.

## Supplementary Material



## Notes

Classical GCS model results in (Figures S1 and S2); the DPFT model results for the concentration effect on the interfacial permittivity in (Figure S3); the comparison between experiments and the DPFT model at 100 mM in (Figure S4); additional analysis by testing an alternative model, DPFT_desol_only, where the potential-dependent solvent-metal interactions were omitted while retaining the potential-dependent partial desolvation of ions effect, in (Figure S5); improved agreement with experiments from DPFT to DPFT_desol in (Figure S6); electrolyte effects on the EDL in (Figure S7); ion desolvation at highly charged states in (Figure S8); the detailed derivations of equations in the main text; basic model parameters in (Table S1); parameters in the GCS models in (Table S2); parameters in the DPFT model in (Table S3); parameters in the DPFT_sol and DPFT_desol model in (Table S4) (PDF)
