# Supplementary material for: Microstructure of Electrical Double Layers at Highly Charged States
Source: JACS Au. 2025 Jun 17;5(7):3453–67. doi: 10.1021/jacsau.5c00508 (PMC12308414; doi:10.1021/jacsau.5c00508)
Supplement: Supplementary file 1 [file au5c00508_si_001.pdf]

Supporting Information to

Microstructure of Electrical Double Layers at Highly Charged States

Zengming. Zhang[1, 2], Jun Huang[1, 2, *]

[1] *Institute of Energy Technologies, IET-3: Theory and Computation of Energy Materials, Forschungszentrum Jülich GmbH, 52425 Jülich, Germany*

[2] *Theory of Electrocatalytic Interfaces, Faculty of Georesources and Materials Engineering, RWTH Aachen University, Aachen 52062, Germany*

* *Corresponding author: ju.huang@fz-juelich.de*

Table of Contents

1. The classical Gouy-Chapman-Stern (GCS) model 2

2. The DPFT model results for the concentration effect on the interfacial permittivity 4

3. The comparison between experiments and the DPFT model at 100mM 5

4. How important is it to include the potential dependent effect of metal-solvent interactions? 5

5. Improved agreement with experiments from DPFT to DPFT_desol 6

6. Electrolyte effects on the EDL 7

7. Ion desolvation at highly charged states 8

8 The detailed derivations of equations (2), (4), (9), and (18) in the manuscript 9

Table S1. Basic model parameters 19

Table S2. Parameters in the GCS model 21

Table S3. Parameters in the DPFT model 21

Table S4. Parameters in the DPFT_sol and DPFT_desol model 23

Reference 24

1. The classical Gouy-Chapman-Stern (GCS) model

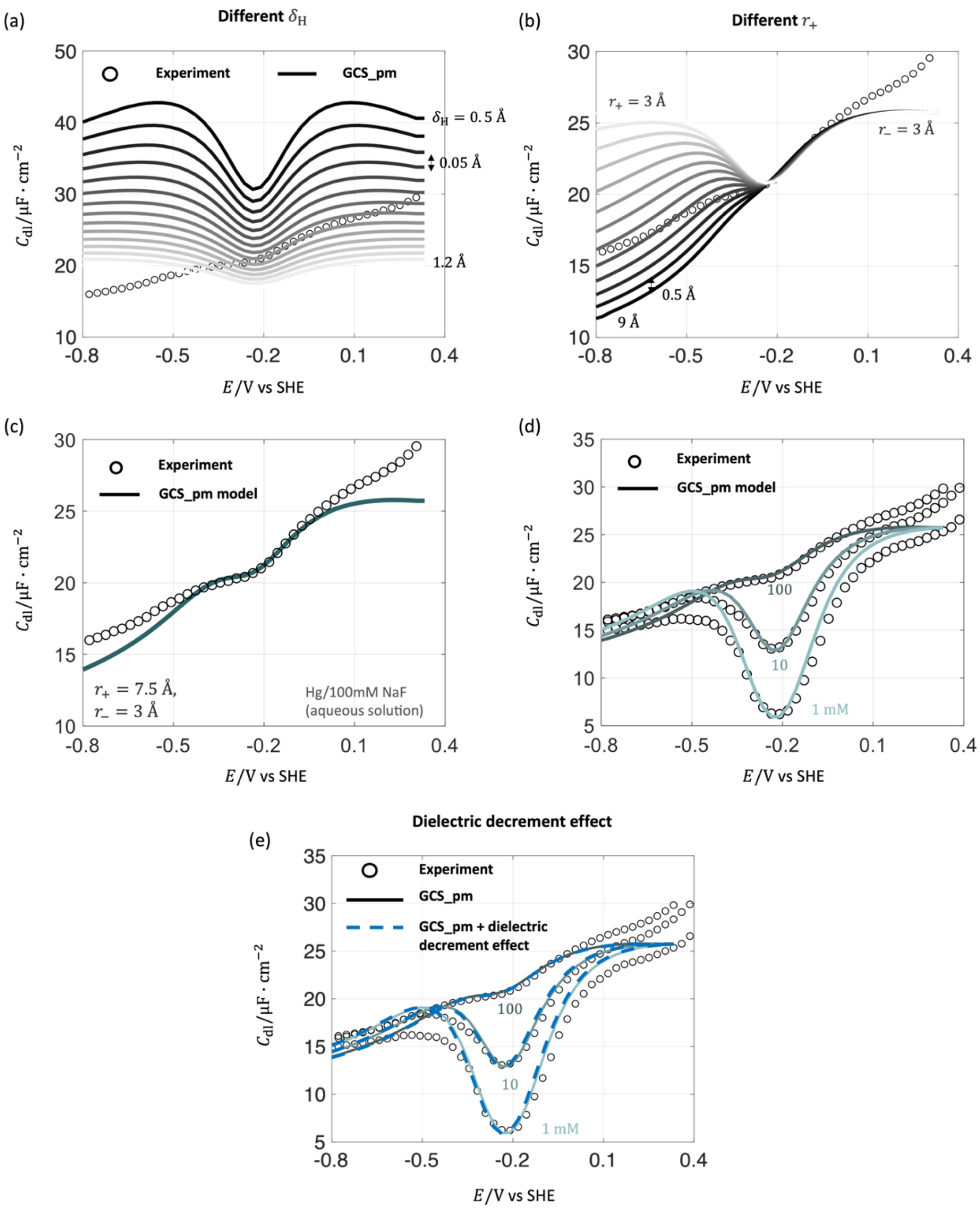

Figure S1. The GCS_pm model[1,2]. (a, b) the GCS_pm model with different δ_H and r_+, respectively. Comparison of C_{dl} between the GCS_pm model and experimental results at (c) 100mM concentration (d) varying ion concentration. (e) Comparison between the GCS_pm model, with and without accounting the dielectric decrement effect, and experimental results at varying ion concentrations. The permittivity values for 1mM, 10mM and 100mM are 78.48, 78.35 and 77.07, respectively, estimated using the empirical equation (16) in ref.[3]. Experimental data were reported by Grahame et al[4]. Fitted parameters are $\varepsilon_H = 6$, $\delta_H = 0.91$ Å, $r_- = 3$ Å, $r_+ = 7.5$ Å. The electric potential is on the SHE scale.

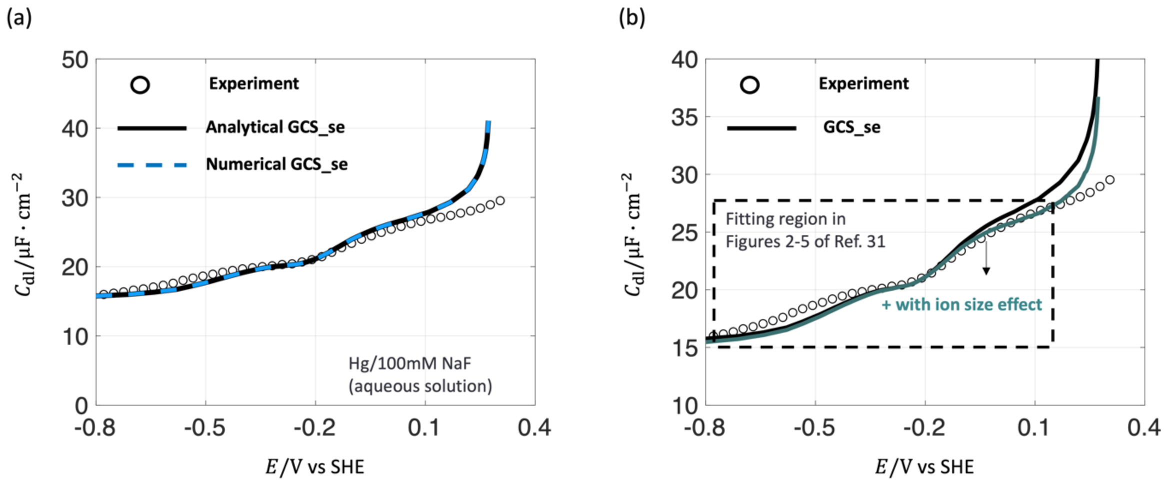

Figure S2. Comparison of C_{dl} between the GCS_se model and experimental results in Hg-100mM NaF aqueous solution. (a) Numerical (dashed line) and analytical GCS_se (solid line) model, (b)The numerical GCS_se model considering the ion size effect. Experimental data were reported by Grahame et al[4]. The electric potential is on the SHE scale.

2. The DPFT model results for the concentration effect on the interfacial permittivity

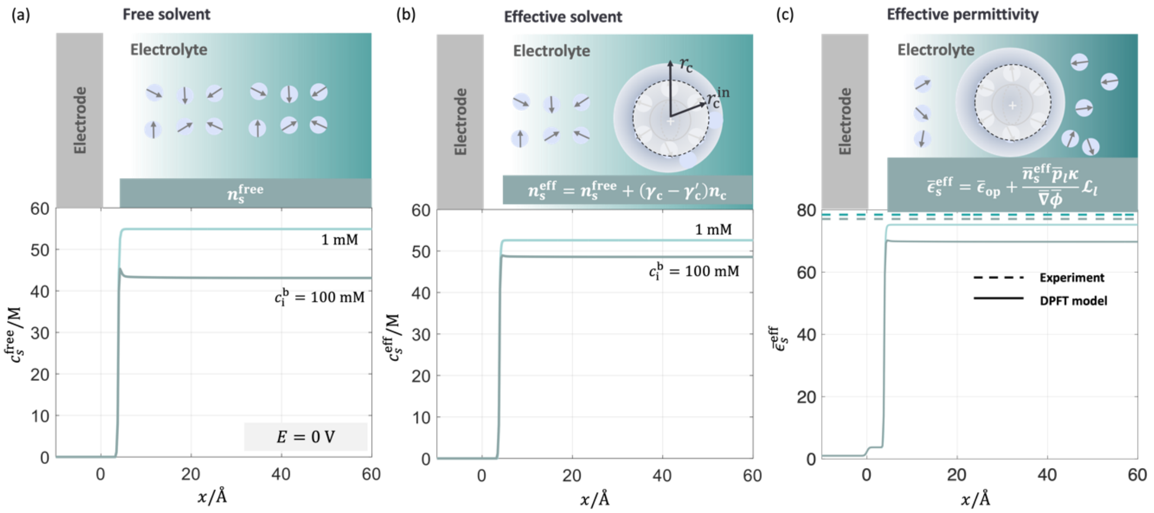

Figure S3. DPFT model results for the Hg-NaF aqueous interface at potential of zero charge (pzc). Concentration effect on the distribution of (a) free solvent density c_s^{free}, (b) effective solvent density c_s^{eff}, and (c) effective interfacial permittivity $\bar{\epsilon}_s^{eff}$ and experimental results are plotted in dashed line as the reference value[3], the value for 1mM and 100mM are 78.48 and 77.07, respectively. The position $x = 0$ denotes the metal edge.

3. The comparison between experiments and the DPFT model at 100mM

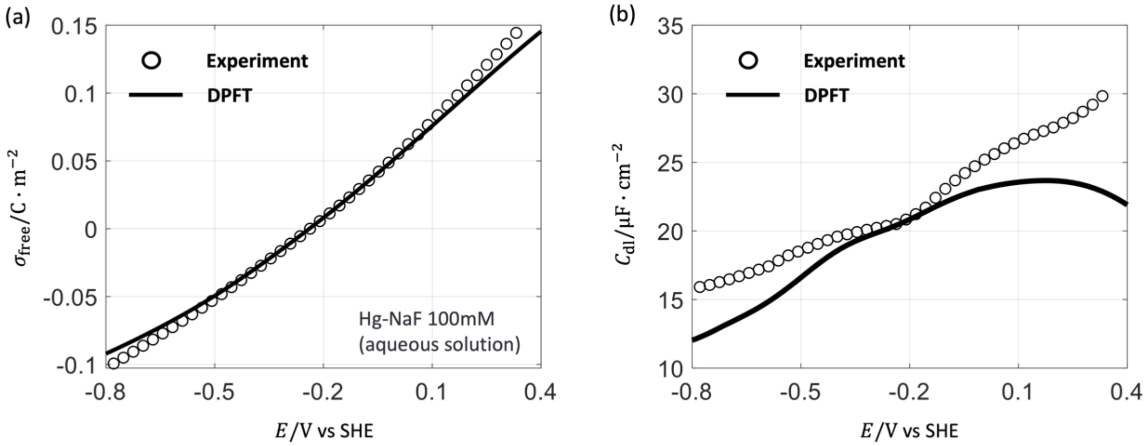

Figure S4. Comparison of (a) σ_{free} and (b) C_{dl} profiles between experimental results (circle) and the DPFT model results (solid line) for the Hg-NaF aqueous solution interface at 100mM.

4. How important is it to include the potential dependent effect of metal-solvent interactions?

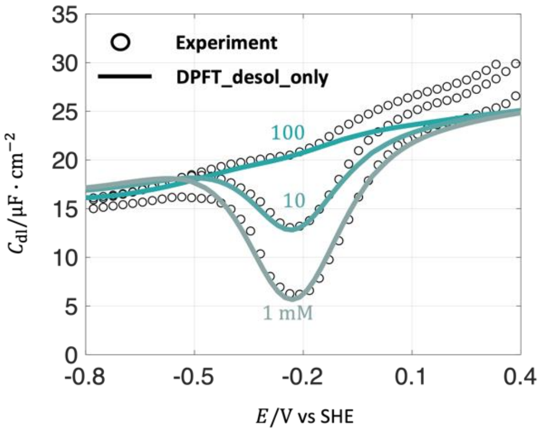

Figure S5. Comparison of C_{dl} between experimental results (circle) and DPFT_desol_only model results (solid line) in Hg-NaF aqueous solution at varying ion concentrations. The accordingly fitting parameter are $\alpha_{\mathrm{ms}} = 0, \beta_{\mathrm{ms}} = 0$ and $\zeta_{\mathrm{Na}^+} = 0.71, \zeta_{\mathrm{F}^-} = 11.1$. Other model parameters are the same with the DPFT_desol model.

5. Improved agreement with experiments from DPFT to DPFT_desol

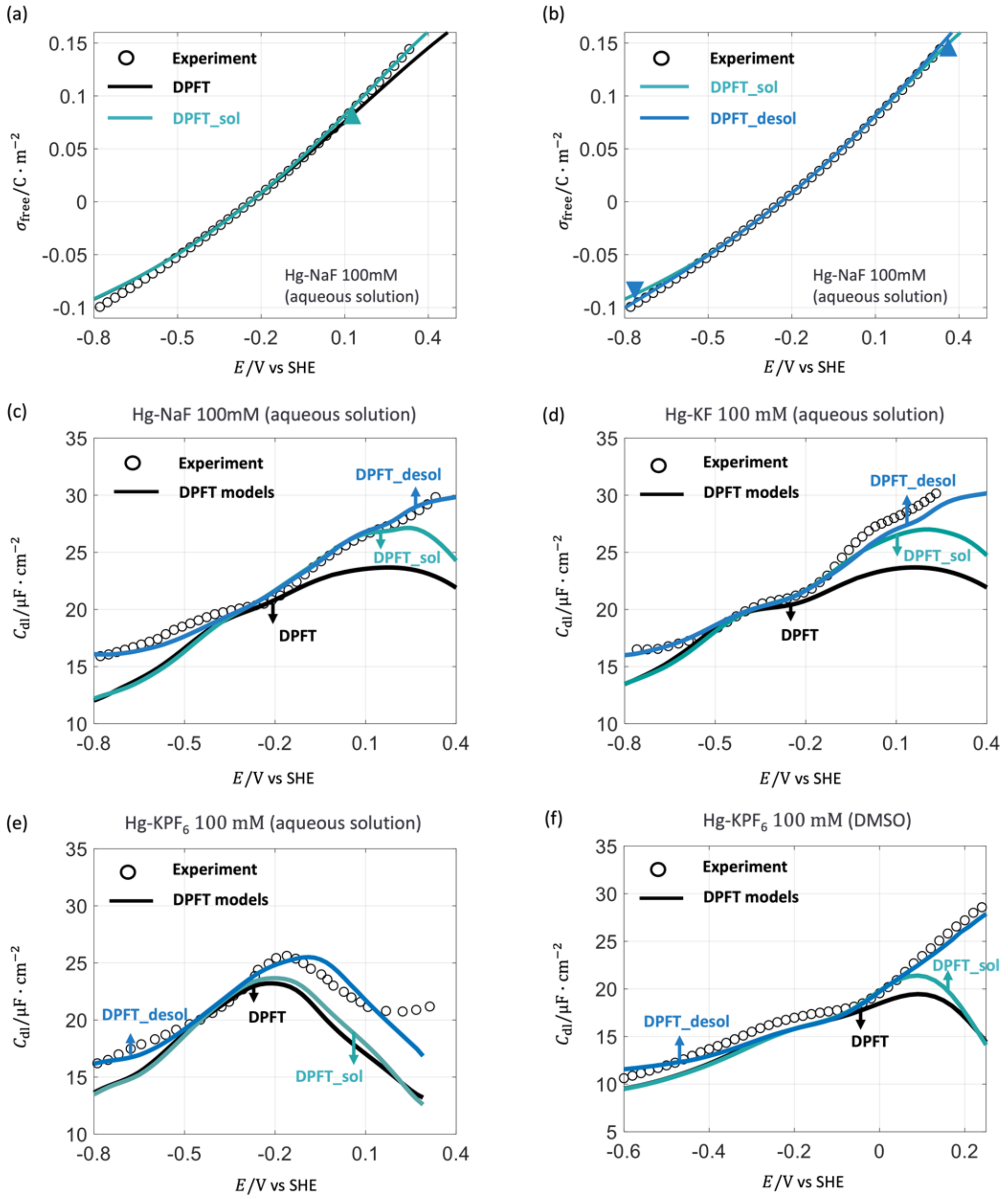

Figure S6. Comparison of σ_{free} (a, b) between experiment and DPFT models for the Hg-NaF aqueous solution interface at 100mM. Comparison of C_{dl} between the DPFT models and experimental results for (c) Hg-KF aqueous solution reported by Grahame et al[4], (d) Hg-KF aqueous solution reported by Schiffrin[5], (e) for Hg-KPF_6 aqueous solution reported by Parsons et al.[6], and (f) for Hg-KPF_6 DMSO solution reported by Payne et al.[7].

6. Electrolyte effects on the EDL

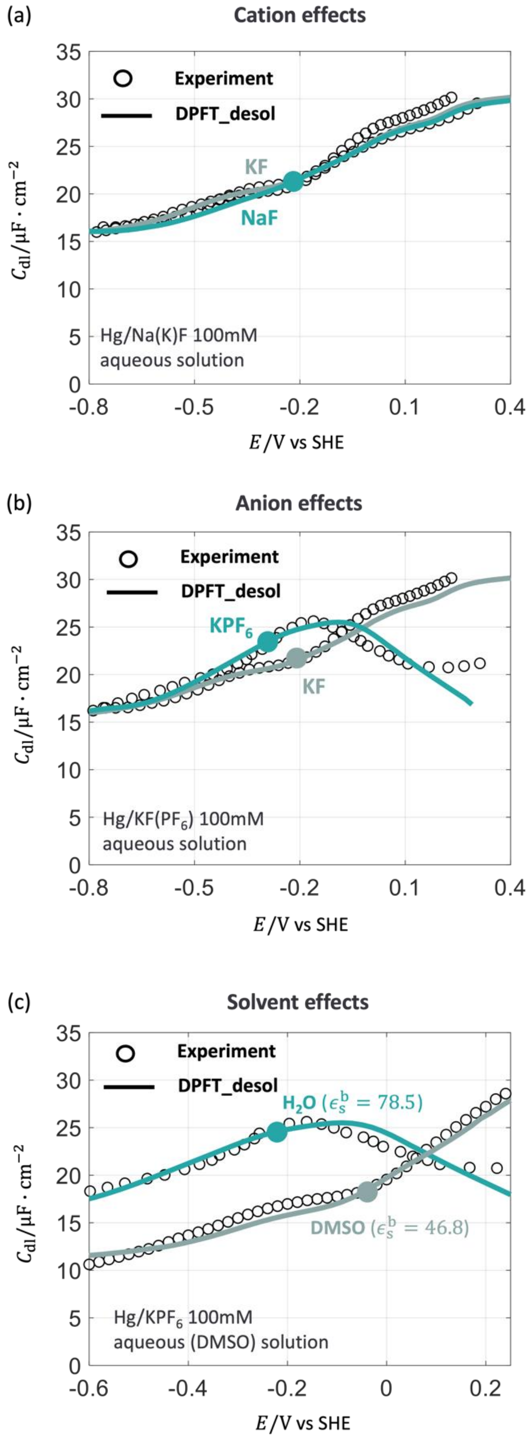

Figure S7. Comparison of C_{dl} between experimental results and the DPFT_desol model at a concentration of 100mM. (a) Effects of cations, Na^+ and K^+, on C_{dl}, (b) Effects of anions, PF_6^- and F^-, on C_{dl}, (c) Effects of solvent molecules, water and DMSO, on C_{dl}.

7. Ion desolvation at highly charged states

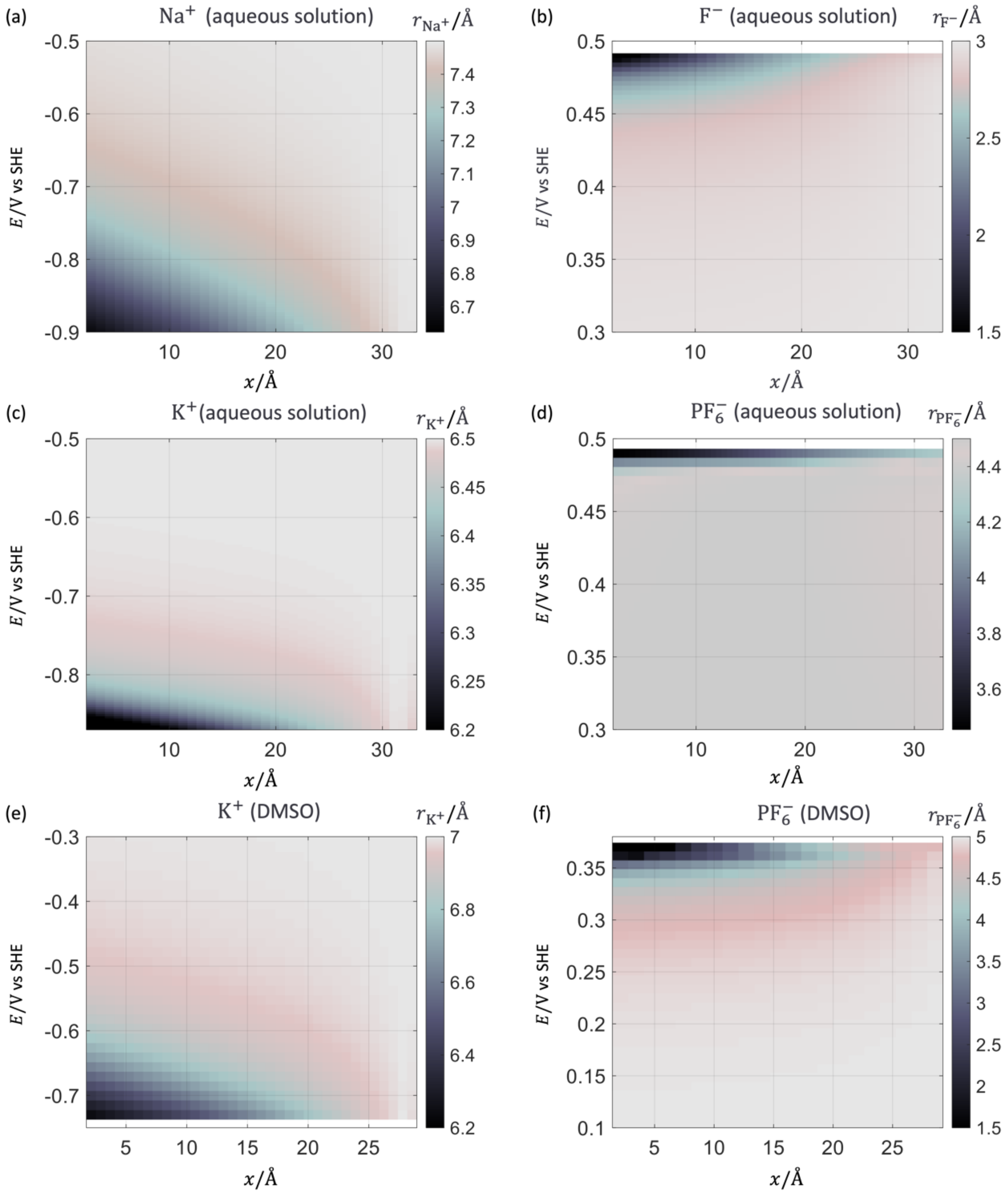

Figure S8. Ion desolvation as function of electrode potential in the DPFT_desol model. The distribution of the radii of solvated ion (a) $Na^{+}(H_2O)$, (b) $F^{-}(H_2O)$, (c) $K^{+}(H_2O)$, (d) $PF_6^{-}(H_2O)$, (e) $K^{+}(DMSO)$, and (f) $PF_6^{-}(DMSO)$ from the metal surface to the solution bulk are as function of electrode potential. The electrode potential is referenced to the SHE scale.

8 The detailed derivations of equations (2), (4), (9), and (18) in the manuscript

1. the detailed derivation of **Equation (2)** in manuscript

The Poisson-Boltzmann (PB) equation describes the distributions of the electric potential and the ion concentrations in the electrolyte solution. Poisson equation reads,

$$\nabla\left(\epsilon_s^{\mathrm{b}}\nabla\phi\right) = -\sum_i z_i F c_i \tag{S1}$$

where ϵ_s^{b} is the bulk dielectric permittivity of the electrolyte solution, ϕ the electric potential, z_i the charge number of ion i, F the Faraday's constant, c_i the concentration of ion i. Boltzmann equation further connects c_i and ϕ,

$$c_i = c_i^{\mathrm{b}} \exp\left(-\frac{z_i F}{RT}\phi\right) \tag{S2}$$

where c_i^{b} is the concentration of ion i in the solution bulk, R the gas constant, T the temperature. For a binary monovalent electrolyte solution in a one-dimensional space, the PB equation is rewritten as,

$$\nabla\left(\epsilon_s^{\mathrm{b}}\nabla\phi\right) = -Fc^{\mathrm{b}}\left(\exp\left(-\frac{F\phi}{RT}\right) - \exp\left(\frac{F\phi}{RT}\right)\right) \tag{S3}$$

where c^{b} the concentration of total anions (cations) in the solution bulk. The dimensionless form of the PB equation is shown as,

$$\frac{\partial^2 U}{\partial X^2} = \sinh(U) \tag{S4}$$

with the dimensionless quantities, $U = F\phi/RT,\ X = x/\lambda_{\mathrm{D}}$, and the Debye length $\lambda_{\mathrm{D}} = \sqrt{\frac{RT\epsilon_{\mathrm{s}}^{\mathrm{b}}\epsilon_0}{2F^2 n_{\mathrm{i}}^{\mathrm{b}}}}$.

The boundary conditions to close Eq. (S4), a second-order differential equation, are,

$$U(X = 0) = U_{\mathrm{HP}} \tag{S5}$$

$$U(X = \infty) = 0 \tag{S6}$$

where $X = 0$ represents the left boundary, at the HP, and $X = \infty$ is the right boundary, in the solution bulk. U_{HP} can be calculated from the electrode side,

$$\phi_{\mathrm{HP}} = E_{\mathrm{M}} - E_{\mathrm{pzc}} + \left(\frac{\partial \phi}{\partial x}\right)_{x=0^+} \frac{\epsilon_{\mathrm{s}}^{\mathrm{b}}}{\epsilon_{\mathrm{HP}}} \delta_{\mathrm{HP}} \tag{S7}$$

where ϵ_{HP} and δ_{HP} are the dielectric permittivity and the thickness of the space between the electrode and the HP, respectively. The coefficient $\epsilon_{\mathrm{s}}^{\mathrm{b}}/\epsilon_{\mathrm{HP}}$ is resultant from the following equality in terms of surface charge density on the electrode surface,

$$\sigma_{\mathrm{M}} = -\epsilon_{\mathrm{s}}^{\mathrm{b}} \left(\frac{\partial \phi}{\partial x}\right)_{x=0^+} = -\epsilon_{\mathrm{HP}} \left(\frac{\partial \phi}{\partial x}\right)_{x=0^-} \tag{S8}$$

Solving Eq. (S4) in the following steps,

$$2 \frac{\partial^2 U}{\partial X^2} \frac{\partial U}{\partial X} = 2\sinh(U) \frac{\partial U}{\partial X} \tag{S9}$$

$$\mathrm{d}\left(\frac{\partial U}{\partial X}\right)^2 = \mathrm{d}(2\cosh U) \tag{S10}$$

$$\left(\frac{\partial U}{\partial X}\right)_{X=0^+}^2 = \left(2\sinh\left(\frac{U_{\mathrm{HP}}}{2}\right)\right)^2 \tag{S11}$$

we obtain the relationship between the excess free surface charge density and the electric potential at the HP,

$$\sigma_{\mathrm{free}} = -\int (c_+ - c_-) F \mathrm{d}x = -\epsilon_{\mathrm{s}}^{\mathrm{b}} \left(\frac{\partial \phi}{\partial x}\right)_{x=0^+} = \frac{2\epsilon_{\mathrm{S}} RT}{F \lambda_{\mathrm{D}}} \sinh\left(\frac{F \phi_{\mathrm{HP}}}{2RT}\right) \tag{S12}$$

The Bikerman-Poisson-Boltzmann (BPB) model treats the electrolyte solution using the lattice-gas approach. Each ion occupies a volume of ${a_{\mathrm{t}}}^3$, where a_{t} is the lattice size. The maximum particle number density is $n_{\mathrm{t}} = {a_{\mathrm{t}}}^{-3}$. The electrochemical potential for ion i reads,

$$\tilde{\mu}_i = \mu_i^0 + z_i e_0 \phi + k_\mathrm{B} T \ln \frac{{a_\mathrm{t}}^3 n_i}{1 - {a_\mathrm{t}}^3 \sum_i \gamma_i n_i} \tag{S13}$$

where μ_i^0 is the chemical potential under standard conditions, e_0 the elementary charge, k_B the Boltzmann constant, n_i the number density of ion i, $\gamma_\mathrm{i} = \left(\frac{2r_\mathrm{i}}{R_\mathrm{s}}\right)^3$ is the relative size of ions referenced to solvent with r_i being the radius of solvated ion and R_s the diameter of solvent. $(1 - {a_\mathrm{t}}^3 \sum_i \gamma_i n_i)/{a_\mathrm{t}}^3$ the number density of solvent molecules. For a binary monovalent electrolyte solution, we have $n_\mathrm{a}^0 = n_\mathrm{c}^0 = n_0^\mathrm{b}$, with n_0^b the number density of total anions (cations) in the solution bulk. Under equilibrium conditions, the electrochemical potential for ion i is uniform in the whole EDL,

$$\tilde{\mu}_i = \mu_i^0 + z_i e_0 \phi + k_\mathrm{B} T \ln \frac{{a_\mathrm{t}}^3 n_i}{1 - {a_\mathrm{t}}^3 \sum_i \gamma_i n_i} = \mu_i^0 + k_\mathrm{B} T \ln \frac{{a_\mathrm{t}}^3 n_0^\mathrm{b}}{1 - {a_\mathrm{t}}^3 \sum_i \gamma_i n_0^\mathrm{b}} \tag{S14}$$

The number density of ion i is obtained as,

$$n_\mathrm{c/a} = \frac{n_0^\mathrm{b} \exp\left(\frac{\mp z_i e_0 \phi}{k_\mathrm{B} T}\right)}{1 + \frac{v}{2}\left(\gamma_\mathrm{c} \exp\left(\frac{-z_i e_0 \phi}{k_\mathrm{B} T}\right) + \gamma_\mathrm{a} \exp\left(\frac{z_i e_0 \phi}{k_\mathrm{B} T}\right) - \gamma_\mathrm{c} - \gamma_\mathrm{a}\right)} \tag{S15}$$

where the bulk volume fraction of solvated ions is $v = 2{a_\mathrm{t}}^3 n_0^\mathrm{b}$. The GCS model assumes $v = 0$.

Combining Eq. (S1) and Eq. (S15), the BPB model is described as,

$$\nabla \cdot \left(\epsilon_\mathrm{s}^\mathrm{b} \nabla \phi\right) = \frac{2 n_0^\mathrm{b} z_i e_0 \sinh\left(\frac{\mp z_i e_0 \phi}{k_\mathrm{B} T}\right)}{1 + \frac{v}{2}\left(\gamma_\mathrm{c} \exp\left(\frac{-z_i e_0 \phi}{k_\mathrm{B} T}\right) + \gamma_\mathrm{a} \exp\left(\frac{z_i e_0 \phi}{k_\mathrm{B} T}\right) - \gamma_\mathrm{c} - \gamma_\mathrm{a}\right)} \tag{S16}$$

The dimensionless form is,

$$\frac{\partial^2 U}{\partial X^2} = \frac{\sinh U}{1 + \frac{v}{2}\left(\gamma_\mathrm{c} e^{-U} + \gamma_\mathrm{a} e^{U} - \gamma_\mathrm{c} - \gamma_\mathrm{a}\right)} \tag{S17}$$

And then the diffuse layer capacitance, C_GC, can be obtained from solving the modified PB equation considering the ion size effect,

$$\boldsymbol{C}_{\mathbf{GC}} = \frac{\partial \boldsymbol{\sigma}_{\mathbf{free}}}{\partial \boldsymbol{U}_{\mathbf{HP}}} = -\frac{\partial}{\partial \boldsymbol{U}_{\mathbf{HP}}}\left(\frac{\partial \boldsymbol{U}}{\partial \boldsymbol{X}}\right)_{\boldsymbol{X}=\mathbf{HP}^{+}} \tag{S18}$$

2. the detailed derivation of **Equation (4)** and **(9)** in manuscript

The volumetric grand potential g of the EDL is written as[8,9],

$$g = e_{\mathrm{au}} a_0^{-3}\left(t_{\mathrm{TF}}(1+\theta_{\mathrm{T}} s^2) + u_{\mathrm{X}}^0(1+\theta_{\mathrm{X}} s^2) + u_{\mathrm{C}}^0 + \theta_{\mathrm{C}} n_{\mathrm{e}} a_0^3 t^2\right)$$

$$+(n_{\mathrm{cc}} - n_{\mathrm{e}}) e_0 \phi - \frac{1}{2}\epsilon_{\mathrm{op}}(\nabla\phi)^2$$

$$+\sum_{l=1}^{N_{\mathbb{C}}} n_l \left(w_l + \delta(l \in M) q_l \phi - \delta(l \in S)\beta^{-1} \ln \frac{\sinh(\beta p_l |\nabla\phi|)}{\beta p_l |\nabla\phi|}\right) \tag{S19}$$

$$+\sum_{l=1}^{N_{\mathbb{C}}} \beta^{-1} n_l \left(\ln\left(n_l \Lambda_l^3\right) - 1\right) + \Phi_{\mathrm{ex}}(\{n_l\}) - n_{\mathrm{e}} \tilde{\mu}_{\mathrm{e}} - \sum_{l=1}^{N_{\mathbb{C}}} n_l \tilde{\mu}_l,$$

which is an orbital-free, hybrid density-potential functional.

Variational analysis

Variational analysis of g in terms of ϕ gives,

$$\frac{\partial g}{\partial \phi} - \nabla \cdot \left(\frac{\partial g}{\partial \nabla\phi}\right) = 0, \tag{S20}$$

leading to,

$$-\nabla[\epsilon_{\mathrm{eff}} \nabla\phi] = e_0(n_{\mathrm{cc}} - n_{\mathrm{e}}) + \sum_{l=1}^{N_{\mathbb{C}}} \delta(l \in \mathrm{M}) n_l q_l, \tag{S21}$$

which is the Poisson equation with an effective dielectric constant as in ref.[9-10]

$$\epsilon_{\mathrm{eff}} = \epsilon_{\mathrm{op}} + \sum_{l=1}^{N_{\mathbb{C}}} \frac{\delta(l \in \mathrm{S}) n_l p_l}{|\nabla\phi|} \left[\coth(\beta p_l |\nabla\phi|) - \frac{1}{\beta p_l |\nabla\phi|}\right]. \tag{S22}$$

Variational analysis of g in terms of particle number densities n_l should be divided into two cases. For the case of electrons, we obtain,

$$\nabla \cdot \left[\frac{\partial(t_{\mathrm{ni}} + u_{\mathrm{X}} + u_{\mathrm{C}})}{\partial \nabla n_{\mathrm{e}}}\right] = \frac{\partial(t_{\mathrm{ni}} + u_{\mathrm{X}} + u_{\mathrm{C}})}{\partial n_{\mathrm{e}}} - e_0\phi - \tilde{\mu}_{\mathrm{e}}. \tag{S23}$$

The electrochemical potential of electrons can be tuned by the electrode potential, ϕ_{M},

$$\tilde{\mu}_{\mathrm{e}} = \mu_{\mathrm{e}} - e_0\phi_{\mathrm{M}}, \tag{S24}$$

with $\mu_{\mathrm{e}} = \frac{\partial t_{\mathrm{TF}}}{\partial \bar{n}_{\mathrm{e}}} + \frac{\partial u_{\mathrm{X}}^0}{\partial \bar{n}_{\mathrm{e}}} + \frac{\partial u_{\mathrm{C}}^0}{\partial \bar{n}_{\mathrm{e}}}$ being the chemical potential of a homogenous electron gas. ϕ is the electric potential.

Eq. (S21) and (S23) constitute the basic set of differential equations controlling the EDL. Next, we need to derive expressions of n_l as functions of ϕ. Variational analysis of g in terms of charged particles in solution gives,

$$w_l + \delta(l \in \mathrm{M}) q_l \phi - \delta(l \in \mathrm{S}) \beta^{-1} \ln\left[\frac{\sinh(\beta p_l |\nabla\phi|)}{\beta p_l |\nabla\phi|}\right]$$
$$+\beta^{-1} \ln\left(n_l \Lambda_l^3\right) + \mu_l^{\mathrm{ex}} - \tilde{\mu}_l = 0, \tag{S25}$$

where μ_l^{ex} is the excess chemical potential, given by,

$$\mu_l^{\mathrm{ex}} = \frac{\delta \Phi_{\mathrm{ex}}}{\delta n_l}. \tag{S26}$$

In this work, the excess term Φ_{ex} is described at the level of Bikerman theory. Bikerman developed a lattice gas approach to calculate the mixing entropy of the electrolyte solution.[11] The Bikerman theory assumes that all charged particles have the same size Λ_{B}.The maximum number density is $n_{\max} = (\Lambda_{\mathrm{B}})^{-3}$. The Bikerman theory gives,

$$\mu_l^{\mathrm{ex}} = \beta^{-1} \ln\left(\frac{1}{1 - \sum_{l=1}^{N_{\mathbb{C}}} n_l \Lambda_{\mathrm{B}}^3}\right) \tag{S27}$$

A more advanced description is the fundamental measure theory (FMT),[12-14], which has been compared with the Bikerman theory in a previous work.[1]

From Eq. (S25), n_l is given by,

$$\frac{n_l \Lambda_l^3}{1 - \sum_{l=1}^{N_{\mathbb{C}}} n_l \Lambda_{\mathrm{B}}^3} = \Theta_l \exp(\beta \tilde{\mu}_l), \tag{S28}$$

where thermodynamic factors can be found as,

$$\boldsymbol{\Theta_l = \exp\left(-\beta\left(\delta(l \in \mathrm{M}) q_l \phi - \delta(l \in \mathrm{S}) \beta^{-1} \ln \frac{\sinh(\beta p_l |\nabla\phi|)}{\beta p_l |\nabla\phi|} + w_l\right)\right)}. \tag{S29}$$

Eq. (S31) shall be valid also in the solution bulk where $\Theta_l = 1$, and $\tilde{\mu}_l$ is uniform in the electrolyte solution. Combining these two conditions, we have the following equality,

$$\frac{n_l \Lambda_l^3}{1 - \sum_{l=1}^{N_{\mathbb{C}}} n_l \Lambda_{\mathrm{B}}^3} = \Theta_l \frac{n_l^b \Lambda_l^3}{1 - \sum_{l=1}^{N_{\mathbb{C}}} n_l^b \Lambda_{\mathrm{B}}^3}. \tag{S30}$$

We have,

$$n_l = n_{\max} \frac{\chi_l \Theta_l}{1 + \sum_{l=1}^{N_{\mathbb{C}}} \chi_l (\Theta_l - 1)}. \tag{S31}$$

with dimensionless bulk number densities $\chi_l = n_l^b / n_{\max}$.

Eq. (S31) can be extended to scenarios of unequal sizes,

$$n_l = n_{\max} \frac{\chi_l \Theta_l}{\Omega}. \tag{S32}$$

where $\Omega = 1 + \sum_{l=1}^{N_{\mathbb{C}}} \gamma_l \chi_l (\Theta_l - 1)$ is the normalization factor, and γ_l is the relative size of particles of type l referenced to Λ_B.

Formal transformation

To facilitate numerical implementation, we manipulate the controlling equations in Eq.(S21) and (S23) further. We can rewrite Eq. (S23) in terms of the dimensionless electron density, $\bar{n}_e = n_\mathrm{e} a_0^3$,

$$\bar{\nabla} \cdot \left[\frac{\partial(t_\mathrm{ni} + u_\mathrm{X} + u_\mathrm{C})}{\partial \bar{\nabla} \bar{n}_e}\right] = \frac{\partial(t_\mathrm{ni} + u_\mathrm{X} + u_\mathrm{C})}{\partial \bar{n}_e} - a_0^{-3}(e_0\phi + \tilde{\mu}_\mathrm{e}) \tag{S33}$$

where the terms are obtained as,

$$\frac{\partial(t_\mathrm{ni} + u_\mathrm{X} + u_\mathrm{C})}{\partial \bar{\nabla} \bar{n}_e} = \frac{\partial(t_\mathrm{ni} + u_\mathrm{X} + u_\mathrm{C})}{\partial s^2} \frac{\partial s^2}{\partial \bar{\nabla} \bar{n}_e}$$
$$= \frac{e_\mathrm{au} a_0^{-3}\left(\theta_\mathrm{T} t_\mathrm{TF} + \theta_\mathrm{XC} u_\mathrm{X}^0\right)}{2(3\pi^2)^{\frac{2}{3}}(\bar{n}_e)^{\frac{8}{3}}} \bar{\nabla} \bar{n}_e, \tag{S34}$$

with $\theta_\mathrm{XC} = \theta_\mathrm{X} - \frac{\pi^2}{3}\theta_\mathrm{C}$, and

$$\frac{\partial(t_\mathrm{ni} + u_\mathrm{X} + u_\mathrm{C})}{\partial \bar{n}_e}$$
$$= e_\mathrm{au} a_0^{-3}[(1 + \theta_\mathrm{T} s^2)\frac{\partial t_\mathrm{TF}}{\partial \bar{n}_e} + (1 + \theta_\mathrm{XC} s^2)\frac{\partial u_\mathrm{X}^0}{\partial \bar{n}_e} + \frac{\partial u_\mathrm{C}^0}{\partial \bar{n}_e} + \left(\theta_\mathrm{T} t_\mathrm{TF} + \theta_\mathrm{X} u_\mathrm{X}^0\right)\frac{\partial s^2}{\partial \bar{n}_e}], \tag{S35}$$

with,

$$\frac{\partial t_{\mathrm{TF}}}{\partial \bar{n}_e} = \frac{1}{2}(3\pi^2)^{\frac{2}{3}}(\bar{n}_e)^{\frac{2}{3}} \tag{S36}$$

$$\frac{\partial s^2}{\partial \bar{n}_e} = -\frac{8}{3}\frac{(\bar{\nabla}\bar{n}_e)^2}{4(3\pi^2)^{\frac{2}{3}}(\bar{n}_e)^{\frac{11}{3}}} = \frac{-8}{3\bar{n}_e}s^2 \tag{S37}$$

$$\frac{\partial u_{\mathrm{X}}^0}{\partial \bar{n}_e} = -\left(\frac{3}{\pi}\right)^{\frac{1}{3}}(\bar{n}_e)^{\frac{1}{3}} \tag{S38}$$

$$\frac{\partial u_{\mathrm{C}}^0}{\partial \bar{n}_e} = -2\alpha_1(1+\alpha_2 r_s)\ln\left(1+\frac{1}{\xi}\right) - 2\alpha_1\bar{n}_e\left(-\frac{1}{3}\left(\frac{3}{4\pi}\right)^{\frac{1}{3}}(\bar{n}_e)^{-\frac{4}{3}}\right)\cdot$$

$$\left(\alpha_2\ln\left(1+\frac{1}{\xi}\right) - \frac{(1+\alpha_2 r_s)}{\xi(1+\xi)}\alpha_1\left(\alpha_3 r_s^{-\frac{1}{2}} + 2\alpha_4 + 3\alpha_5 r_s^{\frac{1}{2}} + 4\alpha_6 r_s\right)\right)$$

$$= -2\alpha_1(1+\alpha_2 r_s)\ln\left(1+\frac{1}{\xi}\right) \tag{S39}$$

$$+\frac{2\alpha_1 r_s}{3}\left(\alpha_2\ln\left(1+\frac{1}{\xi}\right) - \frac{\alpha_1(1+\alpha_2 r_s)}{\xi(1+\xi)}\left(\alpha_3 r_s^{-\frac{1}{2}} + 2\alpha_4 + 3\alpha_5 r_s^{\frac{1}{2}} + 4\alpha_6 r_s\right)\right),$$

We expand the term on the right most side of Eq.(S34),

$$\bar{\nabla}\cdot\left[\frac{\left(\theta_{\mathrm{T}}t_{\mathrm{TF}} + \theta_{\mathrm{X}}u_{\mathrm{X}}^0\right)}{(\bar{n}_e)^{\frac{8}{3}}}\bar{\nabla}\bar{n}_e\right] = \frac{\left(\theta_{\mathrm{T}}t_{\mathrm{TF}} + \theta_{\mathrm{XC}}u_{\mathrm{X}}^0\right)}{(\bar{n}_e)^{\frac{8}{3}}}\bar{\nabla}^2\bar{n}_e - \bar{\nabla}\left[\frac{\left(\theta_{\mathrm{T}}t_{\mathrm{TF}} + \theta_{\mathrm{XC}}u_{\mathrm{X}}^0\right)}{(\bar{n}_e)^{\frac{8}{3}}}\right]\cdot\bar{\nabla}\bar{n}_e$$

$$= \frac{\left(\theta_{\mathrm{T}}t_{\mathrm{TF}} + \theta_{\mathrm{XC}}u_{\mathrm{X}}^0\right)}{(\bar{n}_e)^{\frac{8}{3}}}\bar{\nabla}^2\bar{n}_e - (\bar{n}_e)^{-\frac{8}{3}}\left(\theta_{\mathrm{T}}\frac{\partial t_{\mathrm{TF}}}{\partial \bar{n}_e} + \theta_{\mathrm{XC}}\frac{\partial u_{\mathrm{X}}^0}{\partial \bar{n}_e}\right)(\bar{\nabla}\bar{n}_e)^2 \tag{S40}$$

$$-\frac{8}{3}(\bar{n}_e)^{-\frac{11}{3}}\left(\theta_{\mathrm{T}}t_{\mathrm{TF}}+\theta_{\mathrm{XC}}u_{\mathrm{X}}^{0}\right)(\bar{\nabla}\bar{n}_e)^2$$

Combining Eq.(S33),(S34),(S35), we get

$$\bar{\nabla}\bar{\nabla}\bar{n}_e=\frac{2(3\pi^2)^{\frac{2}{3}}(\bar{n}_e)^{\frac{8}{3}}}{\theta_{\mathrm{T}}t_{\mathrm{TF}}+\theta_{\mathrm{X}}'u_{\mathrm{X}}^{0}}[(1+\theta_{\mathrm{T}}s^2)\frac{\partial t_{\mathrm{TF}}}{\partial\bar{n}_e}+(1+\theta_{\mathrm{XC}}s^2)\frac{\partial u_{\mathrm{X}}^{0}}{\partial\bar{n}_e}+\frac{\partial u_{\mathrm{C}}^{0}}{\partial\bar{n}_e}$$

$$+\left(\theta_{\mathrm{T}}t_{\mathrm{TF}}+\theta_{\mathrm{XC}}u_{\mathrm{X}}^{0}\right)\frac{\partial s^2}{\partial\bar{n}_e}-\frac{(e_0\phi+\tilde{\mu}_{\mathrm{e}})}{e_{\mathrm{au}}}] \tag{S41}$$

$$+\frac{8}{3}(\bar{n}_e)^{-1}(\bar{\nabla}\bar{n}_e)^2-\frac{\theta_{\mathrm{T}}\frac{\partial t_{\mathrm{TF}}}{\partial\bar{n}_e}+\theta_{\mathrm{XC}}\frac{\partial u_{\mathrm{X}}^{0}}{\partial\bar{n}_e}}{\theta_{\mathrm{T}}t_{\mathrm{TF}}+\theta_{\mathrm{XC}}u_{\mathrm{X}}^{0}}(\bar{\nabla}\bar{n}_e)^2$$

Substituting Eq.(S37) into Eq.(S41), and using $s=|\bar{\nabla}\bar{n}_e|/\left(2(3\pi^2)^{\frac{1}{3}}(\bar{n}_e)^{\frac{4}{3}}\right)$, we get,

$$\bar{\nabla}^2\bar{n}_e=\frac{2(3\pi^2)^{\frac{2}{3}}(\bar{n}_e)^{\frac{8}{3}}}{\theta_{\mathrm{T}}t_{\mathrm{TF}}+\theta_{\mathrm{XC}}u_{\mathrm{X}}^{0}}\left(\frac{\partial t_{\mathrm{TF}}}{\partial\bar{n}_e}+\frac{\partial u_{\mathrm{X}}^{0}}{\partial\bar{n}_e}+\frac{\partial u_{\mathrm{C}}^{0}}{\partial\bar{n}_e}-\frac{(e_0\phi+\tilde{\mu}_{\mathrm{e}})}{e_{\mathrm{au}}}\right)$$

$$+\left(\frac{4}{3}(\bar{n}_e)^{-1}-\frac{\theta_{\mathrm{T}}\frac{\partial t_{\mathrm{TF}}}{\partial\bar{n}_e}+\theta_{\mathrm{XC}}\frac{\partial u_{\mathrm{X}}^{0}}{\partial\bar{n}_e}}{2(\theta_{\mathrm{T}}t_{\mathrm{TF}}+\theta_{\mathrm{XC}}u_{\mathrm{X}}^{0})}\right)(\bar{\nabla}\bar{n}_e)^2 \tag{S42}$$

Since, $\frac{\partial t_{\mathrm{TF}}}{\partial\bar{n}_e}=\frac{5t_{\mathrm{TF}}}{3\bar{n}_e}$, $\frac{\partial u_{\mathrm{X}}^{0}}{\partial\bar{n}_e}=\frac{4u_{\mathrm{X}}^{0}}{3\bar{n}_e}$, we obtain

$$\frac{4}{3}(\bar{n}_e)^{-1}-\frac{\theta_{\mathrm{T}}\frac{\partial t_{\mathrm{TF}}}{\partial\bar{n}_e}+\theta_{\mathrm{XC}}\frac{\partial u_{\mathrm{X}}^{0}}{\partial\bar{n}_e}}{2(\theta_{\mathrm{T}}t_{\mathrm{TF}}+\theta_{\mathrm{XC}}u_{\mathrm{X}}^{0})}=\frac{\left(\theta_{\mathrm{T}}t_{\mathrm{TF}}+\frac{4}{3}\theta_{\mathrm{XC}}u_{\mathrm{X}}^{0}\right)}{2\bar{n}_e(\theta_{\mathrm{T}}t_{\mathrm{TF}}+\theta_{\mathrm{XC}}u_{\mathrm{X}}^{0})} \tag{S43}$$

At the end of the day, we reformulate the controlling equation for the electron density as,

$$\overline{\nabla}^2\bar{n}_e = \frac{20}{3}\bar{n}_e\frac{\omega}{\theta_T\omega - \theta_{XC}}\left(\frac{\partial t_{TF}}{\partial \bar{n}_e} + \frac{\partial u_X^0}{\partial \bar{n}_e} + \frac{\partial u_C^0}{\partial \bar{n}_e} - \frac{(e_0\phi + \tilde{\mu}_e)}{e_{au}}\right)$$

(S44)

$$+\frac{\left(\theta_T\omega - \frac{4}{3}\theta_{XC}\right)}{2\bar{n}_e(\theta_T\omega - \theta_{XC})}(\overline{\nabla}\bar{n}_e)^2$$

with $\omega = \frac{2}{5}\pi^{\frac{5}{3}}3^{\frac{1}{3}}(\bar{n}_e)^{\frac{1}{3}}$.

3. the detailed derivation of **Equation (18)** in manuscript

The C_{dl} is calculated by differentiating the surface free charge σ_{free} with respect to electrode potential,

$$C_{\mathrm{dl}} = \frac{\partial \sigma_{\mathrm{free}}}{\partial E_{\mathrm{M}}}, \tag{S45}$$

$\tilde{\mu}_e$ is related to the electrode potential E_{M} on the SHE scale according to[10,11],

$$-\tilde{\mu}_e = e_0(E_{\mathrm{M}} + 4.44\ \mathrm{V}) - e_0\chi_s^v, \tag{S46}$$

where χ_s^v is the surface potential at the solution-vacuum interface[12]. We define dimensionless variables, marked with an over-bar, as follows,

$$\bar{n}_l = n_l(a_0)^3, \bar{x} = \frac{x}{a_0}, \bar{\phi} = \frac{e_0\phi}{k_B T}, \bar{p} = \frac{p}{e_0 a}, \bar{q}_l = \frac{q_l}{e_0}, \bar{\epsilon}_{\mathrm{op}} = \frac{\epsilon_{\mathrm{op}}}{\epsilon_0}$$

with ϵ_0 being the vacuum permittivity. We rewrite Equation(S45),

$$C_{\mathrm{dl}} = -e_0\frac{\partial \sigma_{\mathrm{free}}}{\partial \tilde{\mu}_e} = \frac{e_0^2}{a_0^2}\frac{\partial}{\partial \tilde{\mu}_e}\int d\bar{x}(\bar{n}_c - \bar{n}_a) = \frac{e_0^2}{a_0^2}\frac{\partial}{\partial \tilde{\mu}_e}\int d\bar{x}\left(\bar{n}_e - \bar{n}_{cc}^0\right), \tag{S47}$$

with $\sigma_{\mathrm{free}} = -\frac{e_0}{a_0^2}\int d\bar{x}(\bar{n}_c - \bar{n}_a) = -\frac{e_0}{a_0^2}\int d\bar{x}(\bar{n}_e - \bar{n}_{cc}^0)$.

Table S1. Basic model parameters

Category	Symbol	Item	Value	Note
General constants	R	Ideal gas constant	$8.314\ \mathrm{JK^{-1}mol^{-1}}$	
	k_B	Boltzmann constant	$1.38 \cdot 10^{-23}$ J/K	
	T	Temperature	298 K	
	e_0	Elementary charge	$1.6 \times 10^{-19}\mathrm{C}$	
	e_au	Energy constant from arb. units to SI	27.2 eV	
	N_A	Avogadro's number	$6.02 \times 10^{23}/\mathrm{mol}$	
	ϵ_0	Vacuum permittivity	8.85×10^{-11} F/m	
	a_0	Bohr radius	5.29×10^{-11} m	
	n_ref	Reference number density	$(a_0)^{-3}$	
	κ	Dimensionless constant	$e_0^2/(k_\mathrm{B}T\epsilon_0 a_0)$	
Electrolyte	v	Bulk volume fraction of solvated ions	$2{a_0}^3 n_0^\mathrm{b}$	
	$\bar{n}_\mathrm{s,total}^{\mathrm{H_2O}}$	Dimensionless total water number density	$5.5 \times 10^4 N_\mathrm{A}(a_0)^3$	Ref[13]
	$\bar{n}_\mathrm{s,total}^{\mathrm{DMSO}}$	Dimensionless total DMSO number density	$1.41 \times 10^4 N_\mathrm{A}(a_0)^3$	Ref[14]
	R_sol	Diameter of solvent molecule	$1 \times 10^{10} a_0 \left(1/\bar{n}_\mathrm{s,total}\right)^{1/3}$ Å	
	$r_{\mathrm{Na^+}}^{\mathrm{H_2O}}$	Radius of solvated Na^+ in H_2O	7.5 Å	Fitted from experimental data using the GCS_pm model
	$r_{\mathrm{F^-}}^{\mathrm{H_2O}}$	Radius of solvated F^- in H_2O	3 Å	
	$r_{\mathrm{K^+}}^{\mathrm{H_2O}}$	Radius of solvated K^+ in H_2O	6.5 Å	Estimated
	$r_{\mathrm{PF_6^-}}^{\mathrm{H_2O}}$	Radius of solvated PF_6^- in H_2O	4.5 Å	
	$r_{\mathrm{K^+}}^{\mathrm{DMSO}}$	Radius of solvated K^+ in DMSO	7 Å	
	$r_{\mathrm{PF_6^-}}^{\mathrm{DMSO}}$	Radius of solvated PF_6^- in DMSO	5 Å	
	$r_{\mathrm{Na^+}}^{\mathrm{in,H_2O}}$	Inner-layer radius of solvated Na^+ in H_2O	6.0 Å	Estimated[15,16]
	$r_{\mathrm{K^+}}^{\mathrm{in,H_2O}}$	Inner-layer radius of solvated K^+ in H_2O	5.5 Å	
	$r_{\mathrm{K^+}}^{\mathrm{in,DMSO}}$	Inner-layer radius of solvated K^+ in	6.0 Å	

	DMSO		
$\gamma_{c/s}$	Relative size of solvated cations	$(2r_c/R_s)^3$	Ref[17]
$\gamma_{a/s}$	Relative size of solvated anions	$(2r_a/R_s)^3$	
$\epsilon_s^{H_2O}$	Bulk relative permittivity of H_2O	78.5	
ϵ_s^{DMSO}	Bulk relative permittivity of DMSO	46.8	
$\chi_{H_2O}^{v}$	Surface potential of H_2O-vacuum interface	0.13 V	Ref[12]
χ_{DMSO}^{v}	Surface potential of DMSO-vacuum interface	−0.29 V	

Table S2. Parameters in the GCS model

Category	Symbol	Item	Value	Note
Electrolyte	ϵ_{H}	the relative permittivity within the space between HP and metal surface	6	Ref[18]
	δ_{H}	The effective distance between HP and metal surface	0.91 Å	Fitted from experimental data using the GCS_pm model

Table S3. Parameters in the DPFT model

Category	Symbol	Item	Value	Note
Electrolyte	$\bar{\epsilon}_{\mathrm{op}}^{\mathrm{int,H_2O}}$	Optical permittivity of the Hg-aqueous solution interface	3.70	Calibrated with exp. data
	$\bar{\epsilon}_{\mathrm{op}}^{\mathrm{int,DMSO}}$	Optical permittivity of the Hg-DMSO solution interface	2.74	
	p_s	Solvent dipole moment	$\left[\frac{3\left(\varepsilon_{\mathrm{r}}-\epsilon_{\mathrm{op}}^{\mathrm{int}}\right)\epsilon_0 k_{\mathrm{B}}T}{n_{\mathrm{s,total}}N_{\mathrm{A}}}\right]$ D	Ref[17]
	χ_{v}	Volume fraction of vacancy in the bulk solution	0.05	
Metal	$\bar{n}_{\mathrm{cc}}^{0}$	Dimensionless metal electron density of Hg	0.57	
	$\bar{\epsilon}_{\mathrm{op}}^{\mathrm{M}}$	Optical dielectric constant of metal	1	
	θ_{T}	The gradient coefficients tuning the contribution of the semi-local term in kinetic energy	1.53	Calibrated with exp. data
Metal-electrolyte interactions	β_{l}	Coefficient in Morse potential	1	
	D_{ms}^{0}	Force constant of metal-solvent interactions at the pzc	$0.57e_0/k_{\mathrm{B}}T$	Ref[18,19]
	$d_{\mathrm{mH_2O}}^{0}$	Metal-water equilibrium distance at the pzc	3.3 Å	
	$D_{\mathrm{ma(c)}}$	Force constant of metal-ion interactions	$D_{\mathrm{ms}}^{0}/6$	Calibrated with exp. data

$d_{\mathrm{mNa^+}}$	Metal-Na^+ equilibrium distance in water	4 Å
$d_{\mathrm{mK^+}}$	Metal-K^+ equilibrium distance in water	4 Å
$d_{\mathrm{mF^-}}$	Metal-F^- equilibrium distance in water	4 Å
$d_{\mathrm{mPF_6^-}}$	Metal-PF_6^- equilibrium distance in water	3.2 Å
d_{mDMSO}^{0}	Metal-DMSO equilibrium distance at the pzc	2.8 Å
$d_{\mathrm{mK^+}}^{\mathrm{DMSO}}$	Metal-K^+ equilibrium distance in DMSO	4 Å
$d_{\mathrm{mPF_6^-}}^{\mathrm{DMSO}}$	Metal-PF_6^- equilibrium distance at pzc in DMSO	4 Å

Table S4. Parameters in the DPFT_sol and DPFT_desol model

<table>
<tr><th>Systems</th><th>α_{ms}</th><th>β_{ms}</th><th>ζ_{c}</th><th>ζ_{a}</th></tr>
<tr><td>Hg/NaF H_2O</td><td rowspan="3">0.05</td><td rowspan="3">0.40</td><td>0.71</td><td rowspan="2">1.11</td></tr>
<tr><td>Hg/KF H_2O</td><td rowspan="2">0.34</td></tr>
<tr><td>Hg/KPF_6 H_2O</td><td>0.05</td></tr>
<tr><td>Hg/KPF_6 DMSO</td><td>0.05</td><td>0.15</td><td>0.35</td><td>2.35</td></tr>
</table>

Some important formulates in the DPFT_sol and DPFT_desol model:

DPFT_sol:

$$D_{\mathrm{ms}} = D_{\mathrm{ms}}^{0} + \alpha_{\mathrm{ms}}\left(E - E_{\mathrm{pzc}}\right)e_0,$$

$$d_{\mathrm{ms}} = d_{\mathrm{ms}}^{0} - \beta_{\mathrm{ms}}\left(E - E_{\mathrm{pzc}}\right)e_0,$$

where D_{ms}^{0} and d_{ms}^{0} are the well depth and the equilibrium distance between the solvent molecule and the metal surface at the pzc, respectively.

DPFT_desol:

$$\frac{\gamma_{\mathrm{i}}}{\gamma_{\mathrm{i}}^{0}} = 1 - \zeta_{\mathrm{i}}(\bar{\nabla}\bar{\phi}),$$

where γ_{i}^{0} is the relative size of solvated ion in solution bulk, ζ_{i} is a relatively dimensionless coefficient indicating the degree of partial desolvation of ion.

Reference

(1) Kornyshev, A. A. Double-Layer in Ionic Liquids: Paradigm Change? *J. Phys. Chem. B* **2007**, *111* (20), 5545–5557. https://doi.org/10.1021/jp067857o.

(2) Zhang, L.-L.; Li, C.-K.; Huang, J. A Beginners' Guide to Modelling of Electric Double Layer under Equilibrium, Nonequilibrium and AC Conditions. *J Electrochem* **2022**, *28* (2), 4. https://electrochem.xmu.edu.cn/CN/10.13208/j.electrochem.210847.

(3) Valiskó, M.; Boda, D. The Effect of Concentration- and Temperature-Dependent Dielectric Constant on the Activity Coefficient of NaCl Electrolyte Solutions. *J. Chem. Phys.* **2014**, *140* (23), 234508. https://doi.org/10.1063/1.4883742.

(4) Grahame, D. C. Differential Capacity of Mercury in Aqueous Sodium Fluoride Solutions. I. Effect of Concentration at 25°. *J. Am. Chem. Soc.* **1954**, *76* (19), 4819–4823. https://doi.org/10.1021/ja01648a014.

(5) Schiffrin, D. J. Specific Adsorption of Fluoride Ions on Mercury and the Structure of the Mercury/Solutions Interface. *Trans. Faraday Soc.* **1971**, *67*, 3318–3342. https://doi.org/10.1039/tf9716703318.

(6) Baugh, L. M.; Parsons, R. The Adsorption of Potassium Hexafluorophosphate at the Mercury-Water Interface. *J Electroanal Chem* **1972**, *40* (2), 407–417. https://doi.org/10.1016/S0022-0728(72)80386-3.

(7) Payne, Richard. The Electrical Double Layer in Dimethyl Sulfoxide Solutions. *J. Am. Chem. Soc.* **1967**, *89* (3), 489–496. https://doi.org/10.1021/ja00979a004.

(8) Huang, J. Density-Potential Functional Theory of Electrochemical Double Layers: Calibration on the Ag(111)-KPF_6 System and Parametric Analysis. *J. Chem. Theory Comput.* **2023**, *19* (3), 1003–1013. https://doi.org/10.1021/acs.jctc.2c00799.

(9) Bruch, N.; Eikerling, M.; Huang, J. Density-Potential Functional Theory of Metal-Solution Interfaces. In *Encyclopedia of Solid-Liquid Interfaces*; Elsevier, 2024; pp 308–331. https://doi.org/10.1016/B978-0-323-85669-0.00138-0.

(10) Cheng, J.; Sprik, M. Alignment of Electronic Energy Levels at Electrochemical Interfaces. *Phys. Chem. Chem. Phys.* **2012**, *14* (32), 11245–11267. https://doi.org/10.1039/C2CP41652B.

(11) Le, J.-B.; Cheng, J. Modeling Electrochemical Interfaces from Ab Initio Molecular Dynamics: Water Adsorption on Metal Surfaces at Potential of Zero Charge. *Curr. Opin. Electrochem.* **2020**, *19*, 129–136. https://doi.org/10.1016/j.coelec.2019.11.008.

(12) Trasatti, S. Interfacial Behaviour of Non-Aqueous Solvents. *Electrochimica Acta* **1987**, *32* (6), 843–850. https://doi.org/10.1016/0013-4686(87)87072-X.

(13) Gongadze, E.; Velikonja, A.; Perutkova, Š.; Kramar, P.; Maček-Lebar, A.; Kralj-Iglič, V.; Iglič, A. Ions and Water Molecules in an Electrolyte Solution in Contact with Charged and Dipolar Surfaces. *Electrochimica Acta* **2014**, *126*, 42–60. https://doi.org/10.1016/j.electacta.2013.07.147.

(14) Tang, W.; Zhao, S.; Huang, J. Origin of Solvent Dependency of the Potential of Zero Charge. *JACS Au* **2023**, *3* (12), 3381–3390. https://doi.org/10.1021/jacsau.3c00552.

(15) Nightingale, E. R. Jr. Phenomenological Theory of Ion Solvation. Effective Radii of Hydrated Ions. *J. Phys. Chem.* **1959**, *63* (9), 1381–1387. https://doi.org/10.1021/j150579a011.

(16) Rowley, C. N.; Roux, B. The Solvation Structure of Na^+ and K^+ in Liquid Water Determined from High Level *Ab Initio* Molecular Dynamics Simulations. *J. Chem. Theory Comput.* **2012**, *8* (10), 3526–3535. https://doi.org/10.1021/ct300091w.

(17) Huang, J.; Chen, S.; Eikerling, M. Grand-Canonical Model of Electrochemical Double Layers from a Hybrid Density–Potential Functional. *J. Chem. Theory Comput.* **2021**, *17* (4), 2417–2430. https://doi.org/10.1021/acs.jctc.1c00098.

(18) Böcker, J.; Gurskii, Z.; Heinzinger, K. Structure and Dynamics at the Liquid Mercury−Water Interface. *J. Phys. Chem.* **1996**, *100* (36), 14969–14977. https://doi.org/10.1021/jp961065k.

(19) Sellers, H.; Sudhakar, P. V. The Interaction between Water and the Liquid-Mercury Surface. *J. Chem. Phys.* **1992**, *97* (9), 6644–6648. https://doi.org/10.1063/1.463668.
